# An antibody class with a common CDRH3 motif broadly neutralizes sarbecoviruses

**DOI:** 10.1126/scitranslmed.abn6859

**Published:** 2022-04-19

**Authors:** Lihong Liu, Sho Iketani, Yicheng Guo, Ryan G. Casner, Eswar R. Reddem, Manoj S. Nair, Jian Yu, Jasper F-W. Chan, Maple Wang, Gabriele Cerutti, Zhiteng Li, Nicholas C. Morano, Candace D. Castagna, Laura Corredor, Hin Chu, Shuofeng Yuan, Vincent Kwok-Man Poon, Chris Chun-Sing Chan, Zhiwei Chen, Yang Luo, Marcus Cunningham, Alejandro Chavez, Michael T. Yin, David S. Perlin, Moriya Tsuji, Kwok-Yung Yuen, Peter D. Kwong, Zizhang Sheng, Yaoxing Huang, Lawrence Shapiro, David D. Ho

**Affiliations:** ^1^ Aaron Diamond AIDS Research Center, Columbia University Vagelos College of Physicians and Surgeons, New York, NY 10032, USA; ^2^ Department of Microbiology and Immunology, Columbia University Vagelos College of Physicians and Surgeons, New York, NY 10032, USA; ^3^ Zuckerman Mind Brain Behavior Institute, Columbia University, New York, NY 10027, USA; ^4^ Department of Biochemistry and Molecular Biophysics, Columbia University Vagelos College of Physicians and Surgeons, New York, NY 10032, USA; ^5^ State Key Laboratory of Emerging Infectious Diseases, Carol Yu Centre for Infection, Department of Microbiology, Li Ka Shing Faculty of Medicine, The University of Hong Kong, Hong Kong Special Administrative Region, China; ^6^ Centre for Virology, Vaccinology and Therapeutics, Health@InnoHK, Hong Kong Special Administrative Region, China; ^7^ Institute of Comparative Medicine, Columbia University Irving Medical Center, New York, NY 10032, USA; ^8^ AIDS Institute, Li Ka Shing Faculty of Medicine, The University of Hong Kong, Hong Kong Special Administrative Region, China; ^9^ Hackensack Meridian Health Center for Discovery and Innovation, Nutley, NJ 07110, USA; ^10^ Hackensack Meridian School of Medicine, Nutley, NJ 07110, USA; ^11^ Department of Pathology and Cell Biology, Columbia University Vagelos College of Physicians and Surgeons, New York, NY 10032, USA; ^12^ Division of Infectious Diseases, Department of Medicine, Columbia University Vagelos College of Physicians and Surgeons, New York, NY 10032, USA; ^13^ Vaccine Research Center, National Institutes of Health, Bethesda, MD 20892, USA

## Abstract

The devastation caused by severe acute respiratory syndrome coronavirus 2 (SARS-CoV-2) has made clear the importance of pandemic preparedness. To address future zoonotic outbreaks due to related viruses in the sarbecovirus subgenus, we identified a human monoclonal antibody, 10-40, that neutralized or bound all sarbecoviruses tested in vitro and protected against SARS-CoV-2 and SARS-CoV in vivo. Comparative studies with other receptor-binding domain (RBD)-directed antibodies showed 10-40 to have the greatest breadth against sarbecoviruses, suggesting that 10-40 is a promising agent for pandemic preparedness. Moreover, structural analyses on 10-40 and similar antibodies not only defined an epitope cluster in the inner face of the RBD that is well-conserved among sarbecoviruses, but also uncovered a distinct antibody class with a common CDRH3 motif. Our analyses also suggested that elicitation of this class of antibodies may not be overly difficult, an observation that bodes well for the development of a pan-sarbecovirus vaccine.

## Teaser

A monoclonal antibody that neutralizes or binds all sarbecoviruses tested is representative of a reproducible antibody class.

## INTRODUCTION

The coronavirus disease 2019 (COVID-19) pandemic is caused by severe acute respiratory syndrome coronavirus 2 (SARS-CoV-2), which has infected more than 480 million people and resulted in more than 6.1 million deaths (*1*). Multiple variants of this virus have emerged, including some capable of increased transmission or antibody evasion (*2*–*4*). Furthermore, the threat of continued zoonotic spillovers warrants the development of interventions that could broadly combat animal coronaviruses with pandemic potential (*5*).

Numerous anti-SARS-CoV-2 monoclonal antibodies (mAbs) have been isolated and characterized, with several demonstrating clinical utility (*6*, *7*). Some have been reported to possess broadly neutralizing activity against not only SARS-CoV-2, but also other sarbecoviruses (*8*–*16*), a viral subgenus containing both SARS-CoV-2 and SARS-CoV (*17*). Such broadly neutralizing mAbs could serve as a therapeutic adjunct for the current pandemic as well as a useful agent in addressing future zoonoses caused by sarbecoviruses. Given the therapeutic potential and vast array of mAbs that have been described to hold such breadth, a side-by-side evaluation of each of these mAbs is needed. Such a study would be valuable to understand the differing specificities of these mAbs and identify which would best serve as pandemic preparedness agents. Furthermore, identification of commonalities and deficiencies among mAbs would aid in designing antigens for use as a pan-sarbecovirus vaccine.

Here, we report the isolation of three mAbs that broadly neutralize sarbecoviruses. In addition to virological and structural studies of these three mAbs, we conduct a comprehensive comparative analysis of these mAbs together with nine other mAbs that have previously been reported to have broad activity. Although most of these antibodies target a similar site within the receptor binding domain of the SARS-CoV-2 S protein and can be classified as class 4 antibodies (*18*), there is variability in the exact epitope and angle of approach. Consequently, we find that the exhibited breadth and potencies among these 12 mAbs greatly differ. One of the mAbs identified in this study, 10-40, was the only mAb to neutralize or bind all sarbecoviruses tested. We found that 10-40 and many other broadly neutralizing mAbs utilize a common motif in their heavy chain complementarity determining region 3 (*CDRH3*) gene, suggesting that it may be possible to elicit such antibodies in a general manner. Finally, 10-40 exhibited in vivo efficacy against both SARS-CoV-2 and SARS-CoV, suggesting its use as a therapeutic agent. Collectively, we not only identify three broadly sarbecovirus neutralizing mAbs, but also present a comparative evaluation of broadly neutralizing mAbs. The information provided herein could aid the development of pan-sarbecovirus antibodies and vaccines.

## RESULTS

### Isolation of broadly neutralizing mAbs against sarbecoviruses.

To isolate mAbs with the desired neutralization breadth, we screened serum samples from convalescent COVID-19 patients for neutralizing activity against a panel of variant viruses. Serum from Patient 10 and Patient 11 potently neutralized all SARS-CoV-2 variants tested as well as SARS-CoV, albeit weakly (**fig. S1**). We then sorted for B.1.351 S trimer-specific memory B cells from the blood of both patients, followed by single-cell RNA-sequencing to determine the paired heavy and light chain sequences of each mAb (**fig. S2**) (*19*). A total of 58 mAbs were isolated and characterized.

**Fig. 1.**
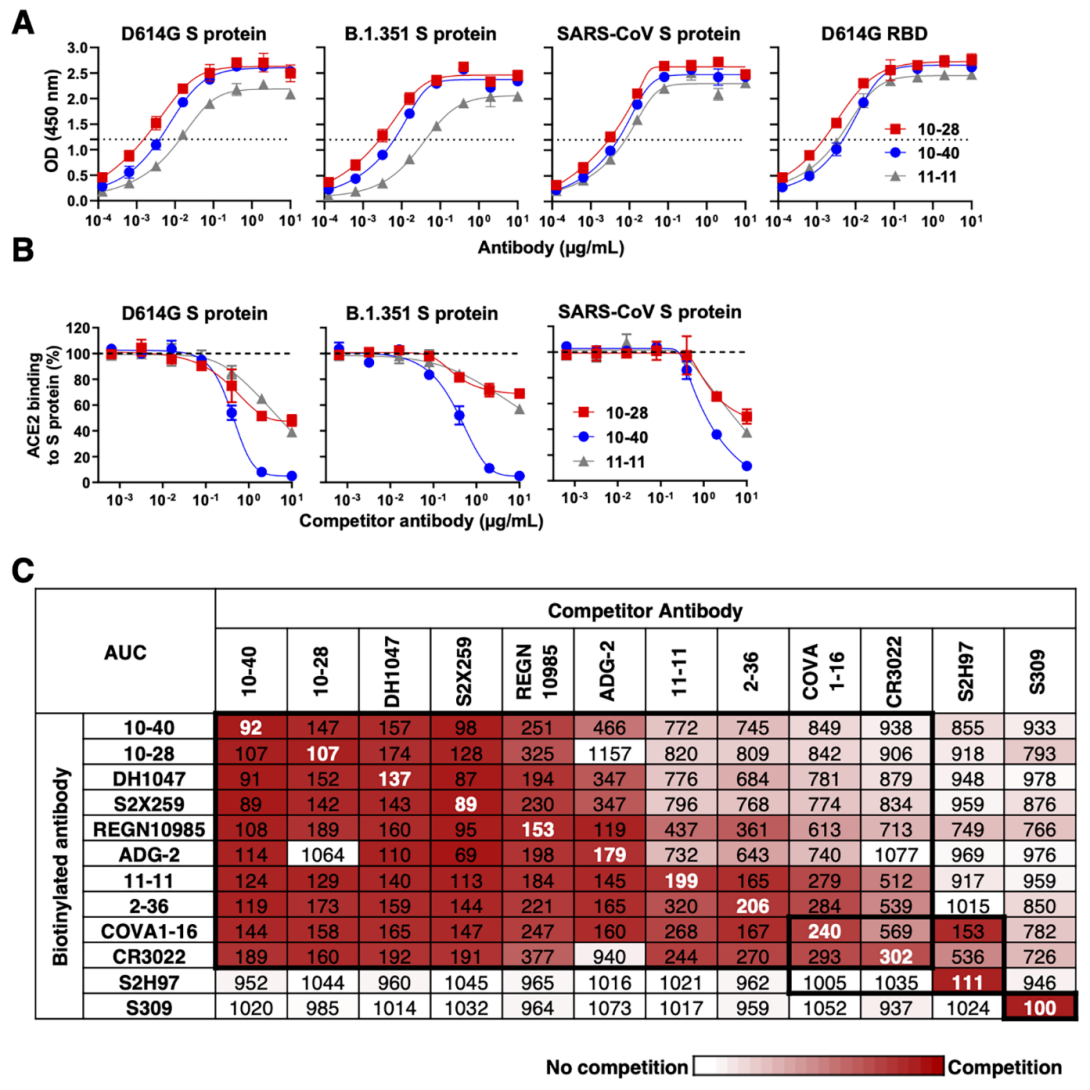
**Three mAbs that bind to the S proteins of SARS-CoV-2 variants and SARS-CoV were isolated from convalescent COVID-19 patients. (A)** 10-28, 10-40, and 11-11 were tested for binding to SARS-CoV-2 D614G, B.1.351, SARS-CoV, as well as the RBD of D614G. OD, optical density. The horizontal dashed lines indicate OD=1.2, the half-maximal binding value. **(B)** Inhibition of ACE2 binding to SARS-CoV-2 D614G, B.1.351, and SARS-CoV S proteins was tested for 10-28, 10-40, and 11-11. Data are shown as mean ± SD of two technical replicates. **(C)** Epitope mapping was done by competition ELISA for 10-28, 10-40, and 11-11 mAbs together with other RBD-directed broadly neutralizing mAbs. A representative result of three experimental replicates is shown. AUC, area under the curve. Boxes with thick black borders indicate antibody clusters.

Three mAbs, 10-40, 10-28, and 11-11, were found to bind to SARS-CoV-2 S proteins of variants D614G and B.1.351 as well as the SARS-CoV S protein (**Fig. 1A**). All three antibodies recognized epitopes within the receptor binding domain (RBD) (**Fig. 1A**) and inhibited the binding of soluble human angiotensin converting enzyme 2 (ACE2) receptor to the S protein (**Fig. 1B**). Epitope mapping by competition enzyme-linked immunosorbent assay (ELISA) was carried out on these three antibodies, along with a panel of nine RBD-specific mAbs reported to have breadth against sarbecoviruses, including DH1047 (*8*), S2X259 (*9*), REGN10985 (*10*), ADG-2 (*11*), 2-36 (*12*), COVA1-16 (*13*), CR3022 (*14*), S2H97 (*15*), and S309, also known as sotrovimab (*16*). 10-40, 10-28, and 11-11 fell into one competition group with seven other mAbs (**Fig. 1C** **and fig. S3**) that are known to recognize an inner face of RBD when it is in the “up” position (*8*–*13*). The epitope of S2H97 was partially overlapping whereas that of S309 was discrete; this was not surprising since the latter is directed to an epitope on the outer face on RBD (*15*, *16*). The binding affinities of this panel of mAbs to SARS-CoV-2 and SARS-CoV S proteins were also measured by surface plasmon resonance **(fig. S4)**.

Genetically, 10-40, 10-28, and 11-11 utilized IGHV4-39*01, IGHV3-30*18, and IGHV4-31*03 heavy chain V (variable) genes with CDRH3 (complementarity determining region) lengths of 22, 13, and 21 amino acids, respectively. The light chains of 10-40, 10-28, and 11-11 were derived from IGLV6-57*01, IGKV1-39*01, and IGLV1-40*01, respectively (**fig. S5A**). All three antibodies had low somatic hypermutation (**fig. S5, A and B**).

### Comparative analysis of mAb neutralization and binding to sarbecoviruses

**Fig. 2.**
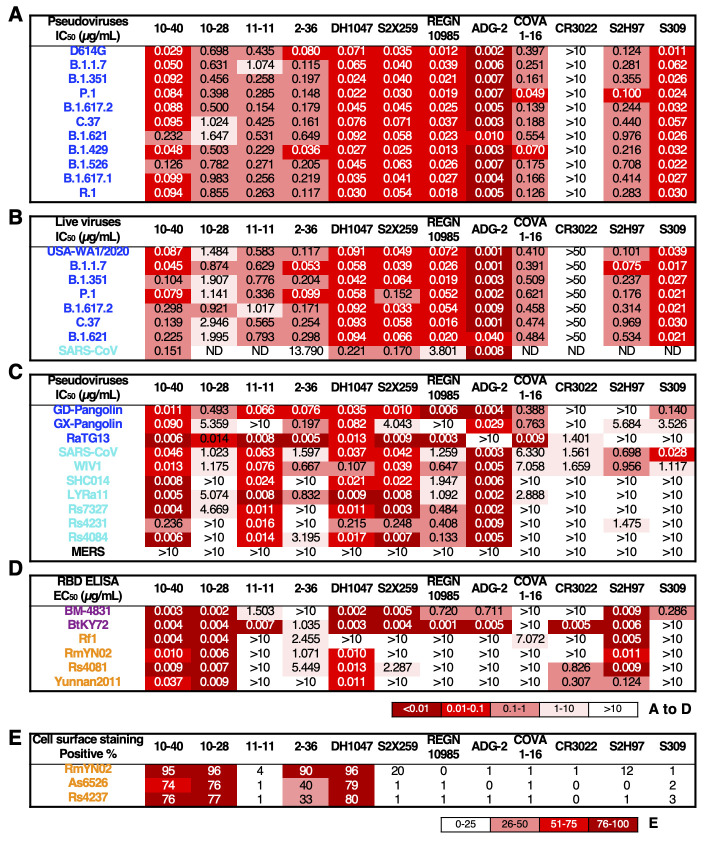
**Breadth and potency of 10-40, 10-28, and 11-11 versus other reported antibodies with broad reactivity. (A to C)** Neutralization titers (IC_50_) are shown for the indicated mAbs against pseudoviruses of SARS-CoV-2 variants **(A)**, authentic SARS-CoV-2 variants and SARS-CoV GZ50 strain **(B)**, and pseudoviruses of other animal sarbecoviruses in the SARS-CoV-2 (blue) and SARS-CoV (green) sublineages **(C). (D)** Binding of mAbs to purified RBD proteins from African and European (purple) or Asian (orange) bat sarbecoviruses was measured by ELISA. EC_50_, 50% effective concentration. **(E)** Binding of mAbs to S proteins expressed on the surface of transfected cells was measured by flow cytometry. A representative result of three experimental replicates is shown. ND, not determined.

We then comprehensively compared the virus-neutralizing potency and breadth of 10-40, 10-28, and 11-11 to other RBD-directed mAbs with known activity against other sarbecoviruses. First, each antibody was evaluated against SARS-CoV-2 variants in neutralization assays using both vesicular stomatitis virus (VSV) ΔG-pseudotyped viruses and authentic viruses. All mAbs, except CR3022, showed breadth by neutralizing all SARS-CoV-2 strains tested (**Fig. 2, A and B** **, fig. S6 and S7A**). ADG-2 was the most potent, followed by a group comprised of 10-40, DH1047, S2X259, REGN10985, and S309. In general, 10-28, 11-11, 2-36, and COVA1-16 exhibited lower potencies. Against authentic SARS-CoV, ADG-2, 10-40, S2X259, and DH1047 showed neutralizing activity with 50% inhibitory concentration (IC_50_) values well below 1 μg/mL (**Fig. 2B** **and fig. S7B**).

We next evaluated each mAb for neutralization against ten non-SARS-CoV-2 sarbecoviruses capable of using human ACE2 as receptor in a VSVΔG-pseudotyped virus assay (**Figs. 2C** **and fig. S8**). Only 10-40 and DH1047 neutralized all sarbecoviruses tested, whereas 11-11, S2X259, and ADG-2 were deficient or weak in neutralizing at least one of the viruses in the panel. The other mAbs had many more “holes” in their repertoire. As expected, none of these antibodies neutralized the Middle East respiratory syndrome virus (MERS), a member of the merbecovirus subgenus.

There are more sarbecoviruses found in bats in Africa and Europe or Asia (**fig. S9A**) that do not use human ACE2 as receptor (*17*, *20*). Since their target cells are unknown, performing virus-neutralization assay is not readily feasible. We therefore examined the binding profiles of this panel of mAbs to RBD proteins derived from six sarbecoviruses outside of SARS-CoV-2 and SARS-CoV sublineages (**Fig. 2D** **and fig. S9B**). 10-40, 10-28, and S2H97 bound all RBDs tested, whereas DH1047 did not recognize the RBD of Rf1. The remaining mAbs bound only a subset of the RBDs. In particular, S2X259, REGN10985, ADG-2, and S309 did not recognize the RBD of most Asian bat sarbecoviruses. Similarly, we assessed the binding of this panel of mAbs to three S proteins derived from Asian bat sarbecoviruses (**fig. S9A**) as expressed on the surface of transfected cells. 10-40, 10-28, 2-36, and DH1047 exhibited binding, but other mAbs were largely non-reactive (**Fig. 2E** **and fig. S10**).

**Fig. 3.**
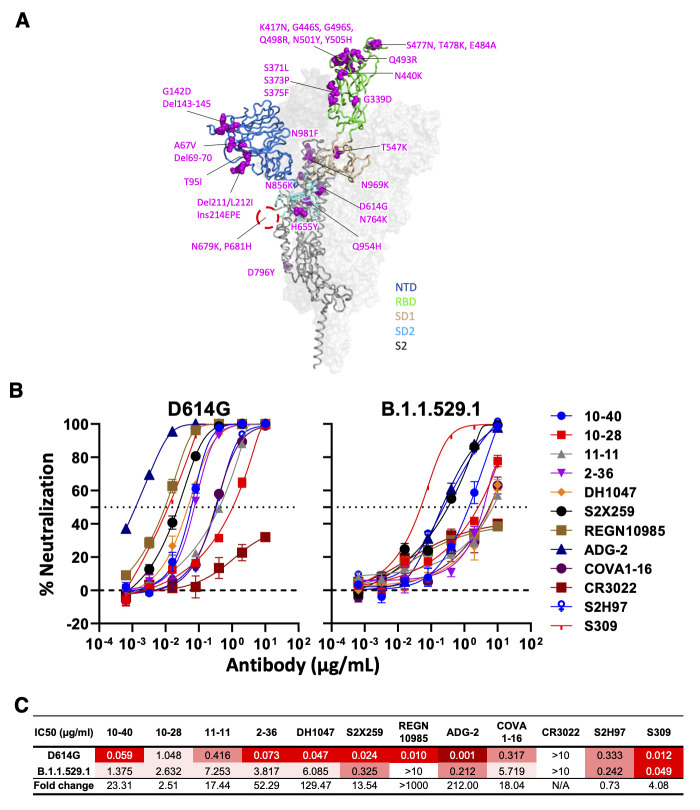
**Neutralizing activity of antibodies varies against the SARS-CoV-2 Omicron variant (BA.1). (A)** Mutations within the Omicron variant, BA.1 (B.1.1.529.1) are denoted on the full SARS-CoV-2 S trimer. The SARS-CoV-2 S protein structure was downloaded from PDB 7KRR. The red circle represents the S1/S2 cleavage site. SD1, subdomain 1; SD2, subdomain 2. **(B)** Neutralization curves of selected mAbs against VSV pseudotypes with D614G (WT) and B.1.1.529.1 S proteins are shown. The dotted horizontal line at 50% indicates IC_50_ values. **(C)** Neutralization titers (IC_50_) of selected mAbs against VSV pseudotypes with D614G (WT) and B.1.1.529.1 S proteins are summarized. Data are shown as mean ± SD of three technical replicates.

Given the recent rise of the Omicron variant of SARS-CoV-2, we placed a particular focus on the how the many mutations found in the variant affected neutralization by the mAbs in our panel (**Fig. 3A**). Previous studies demonstrated that class 4 antibodies were considerably impacted by the Omicron variant, B.1.1.529.1 (also known as BA.1), albeit in a disparate manner (*4*). We expanded on these previous findings by assessing our entire panel of 12 mAbs against B.1.1.529.1 (**Fig. 3, B and C**). All of the mAbs were adversely affected, with the exception of S2H97. Intriguingly, we found that DH1047, REGN10985, and ADG-2 lost over 100-fold potency, whereas others, including 10-28 and S309, only lost a fewfold. 10-40 was moderately impacted, but could still achieve 100% neutralization at the highest dose. As these mAbs are not linked by a shared epitope according to their structures with the wild-type S protein, this suggests that the S protein of B.1.1.529.1 may have a slightly altered conformation in this particular region. In summary, these findings show that 10-40 is a broadly-neutralizing RBD-directed mAb.

### Structural studies of 10-40, 10-28, and 11-11

**Fig. 4.**
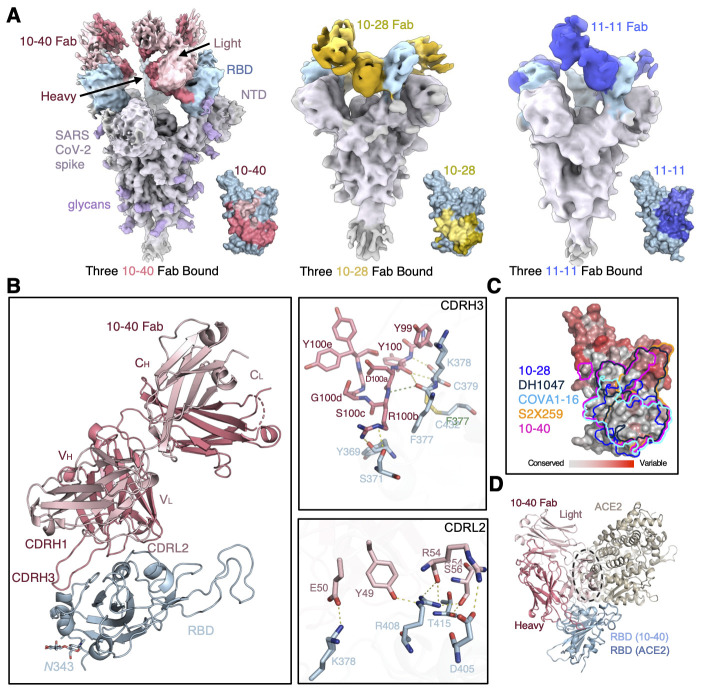
**Structures of isolated antibodies with SARS-CoV-2 S protein and RBD. (A)** Cryo-EM reconstructions and recognition footprints for 10-40, 10-28, and 11-11 Fabs bound to B.1.351 S trimer are shown. The S protein is colored in light gray, with the RBDs in green and the glycans in purple, oriented with the membrane toward the bottom. The 10-40 Fabs are colored in red, the 10-28 Fabs in yellow, and the 11-11 Fabs in dark green. The Fab heavy chains are shaded darker than the light chains. The footprint of each respective antibody on the inner face of RBD is displayed to the right of each S trimer structure. **(B)** The crystal structure of 10-40 Fab bound to WA1 SARS-CoV-2 RBD is shown. The overall structure is shown in the left panel, and specific interactions by CDRH3 and CDRL2 are shown in the right panels, top and bottom, respectively. **(C)** A comparison of 10-28, DH1047, COVA1-16, S2X259, and 10-40 epitope footprints on SARS-CoV-2 RBD is shown. The RBD was colored according to the sequence conservation of each residue across 52 sarbecoviruses. **(D)** An overlay of 10-40 and ACE2 binding to RBD shows a clash between the 10-40 light chain and ACE2, highlighted by the black dashed oval.

To investigate the nature of antibody-S protein interactions for 10-40, 10-28, and 11-11, we determined the cryo-electron microscopy (cryo-EM) structures for Fabs of these mAbs in complex with S2P-prefusion-stabilized S proteins from SARS-CoV-2 WA-1 and B.1.351 strains. We observed a greater degree of S protein disassembly for all WA-1 complexes, and higher quality cryo-EM maps were obtained for all three B.1.351 complexes. A single predominant population was observed where three Fabs were bound per S protein in a 3-RBD-up conformation (**Fig. 4A** **, fig. S11 to S13, table S1**). For 11-11, an additional class of two Fabs bound with 2-RBD up was also observed. The 10-40 complex reconstruction reached 3.5 Å global resolution, but local refinement of the RBD + Fab maps did not surpass 4 Å resolution. To resolve the interfaces, we determined crystal structures of the 10-40 and 10-28 Fab:RBD complexes. Both the crystal structures of 10-40 and 10-28 fitted nearly perfectly in the cryo-EM reconstruction density (**fig. S11 and S12**). For 11-11, a homology model was built and docked into the map (**fig. S13**), which showed this antibody recognizes RBD in the same way as S2X259 (*9*), with both sharing a similar light chain and CDRH3 motif.

To visualize the epitopes of 10-40, 10-28, and 11-11 on the SARS-CoV-2 WA1 RBD at higher resolution, we determined the structures of Fab in complex with RBD using X-ray crystallography, with suitable crystals obtained for 10-40 and 10-28 (**table S2**). The 10-40 crystal structure at 1.5 Å resolution revealed recognition of an epitope that is highly conserved among sarbecoviruses on the inner face of RBD (**Fig. 4, B and C**). 10-40 used three of its six CDR loops, H1, H3, and L2, to interact with an epitope consisting of a loop on RBD (residues 377 to 385) and extended toward the RBD ridge near the ACE2 binding site. 10-40 also established extensive polar contacts and hydrophobic interactions with RBD residues (**Fig. 4B**).

For the 10-28 crystal structure (**fig. S14A**), antibody-RBD side-chain interactions were well defined at 3.2 Å resolution. Interactions were mediated by 10-28 CDR loops H1, H3, L1, and L3, which predominantly contacted α3, but also α2 and the β2-α3, α4-α5 and α5-β4 loops of the RBD. The heavy chain formed three hydrogen bonds and a single salt bridge, between D95 of the CDRH3 and K386 of the RBD, whereas the light-chain residues formed a total of five hydrogen bonds (**fig. S14B**).

All three antibodies recognized a region on the inner side of RBD that is hidden in the RBD-down conformation. Thus, they can only recognize RBD in the up conformation. The 10-40 epitope is similar to the previously defined ‘class 4’ antibody epitope, although we believe all three of these antibodies can be classified as such (*18*). Superposition of the 10-40 Fab with the ACE2-RBD complex (6M0J) showed that antibody binding places the VL domain of the antibody in a position that would clash with ACE2 (**Fig. 4D**), consistent with the experimental data showing inhibition of ACE2 binding (**Fig. 1B**).

Structural comparison of these newly identified antibodies with those previously identified targeting this ‘class 4’ epitope revealed that several modes of binding are available to this site (**fig. S15**) (*8*, *9*, *11*–*14*, *21*–*23*). Similar antibodies were available for each of 10-40, 10-28, and 11-11. Although we observed a relationship between the epitope and the breadth exhibited by these antibodies, there also seems to be additional factors involved, such as the binding angle.

**Fig. 5.**
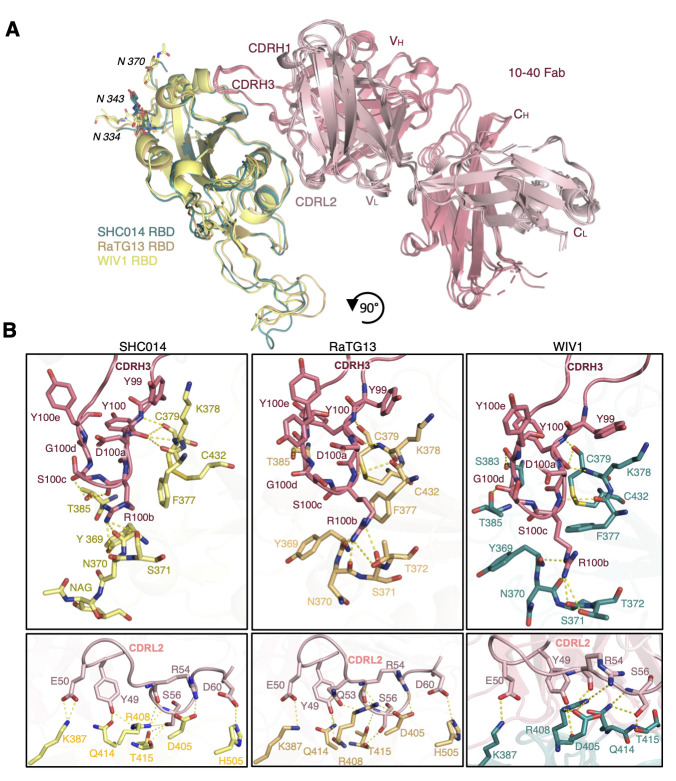
**Structures of sarbecovirus RBD complexes with 10-40. (A)** Superposition of crystal structures with 10-40 Fab bound to RBDs from SHC014 (yellow), RaTG13 (orange), and WIV1 (teal) are shown. 10-40 heavy chain and light chain are colored in red and pink, respectively. Glycans at N334, N343, and N370 are shown. **(B)** Molecular interactions are shown for 10-40 recognition of RBDs from the indicated sarbecoviruses. The orientation is rotated 90° from (A). Dashed lines indicate interactions.

Given the binding breadth exhibited by 10-40 (**Fig. 2**), we conducted additional structural studies for this mAb to investigate the basis for its cross-reactivity. To this end, we determined crystal structures of the 10-40 Fab bound to the RBDs of the sarbecoviruses SHC014, RaTG13, and WIV1 at 2.2 Å, 2.8 Å, and 3.1 Å resolutions, respectively (**Fig. 5, A and B** **, table S3**). These structures demonstrated that 10-40 bound to a highly conserved epitope that allowed for similar binding of 10-40 to these sarbecoviruses and SARS-CoV-2.

As observed in the complex of 10-40 bound to SARS-CoV-2 RBD, the majority of interactions for 10-40 with SHC014, RaTG13, and WIV1 were through its CDRH3 and CDRL2 (**Fig. 4B** **and** **Fig. 5A**). We found that additional hydrogen bonds and salt bridges were present between 10-40 and these three sarbecoviruses as compared to SARS-CoV-2. Specifically, though the 10-40-SARS-CoV-2 interface involved 10 hydrogen bonds and three salt bridges, there were 15, 14, and 17 hydrogen bonds and four, three, and three salt bridges for 10-40 with SHC014, RaTG13, and WIV1, respectively (**Fig. 5B**). These additional contacts may explain why the potency for 10-40 against SHC014, RaTG13, and WIV1 is greater than that for SARS-CoV-2 (**Fig. 2**).

Among the four complexes of 10-40 with sarbecovirus RBDs, there were a total of 12 interactions (nine hydrogen bonds and three salt bridges) that were conserved in all of the complexes (**Fig. 5B**). Such extensive overlap suggests that for mAbs that have deficiencies against some of these sarbecoviruses (**Fig. 2**), engineering of similar interactions could help to improve their breadth.

### Identification of a common motif among broadly neutralizing mAbs

**Fig. 6.**
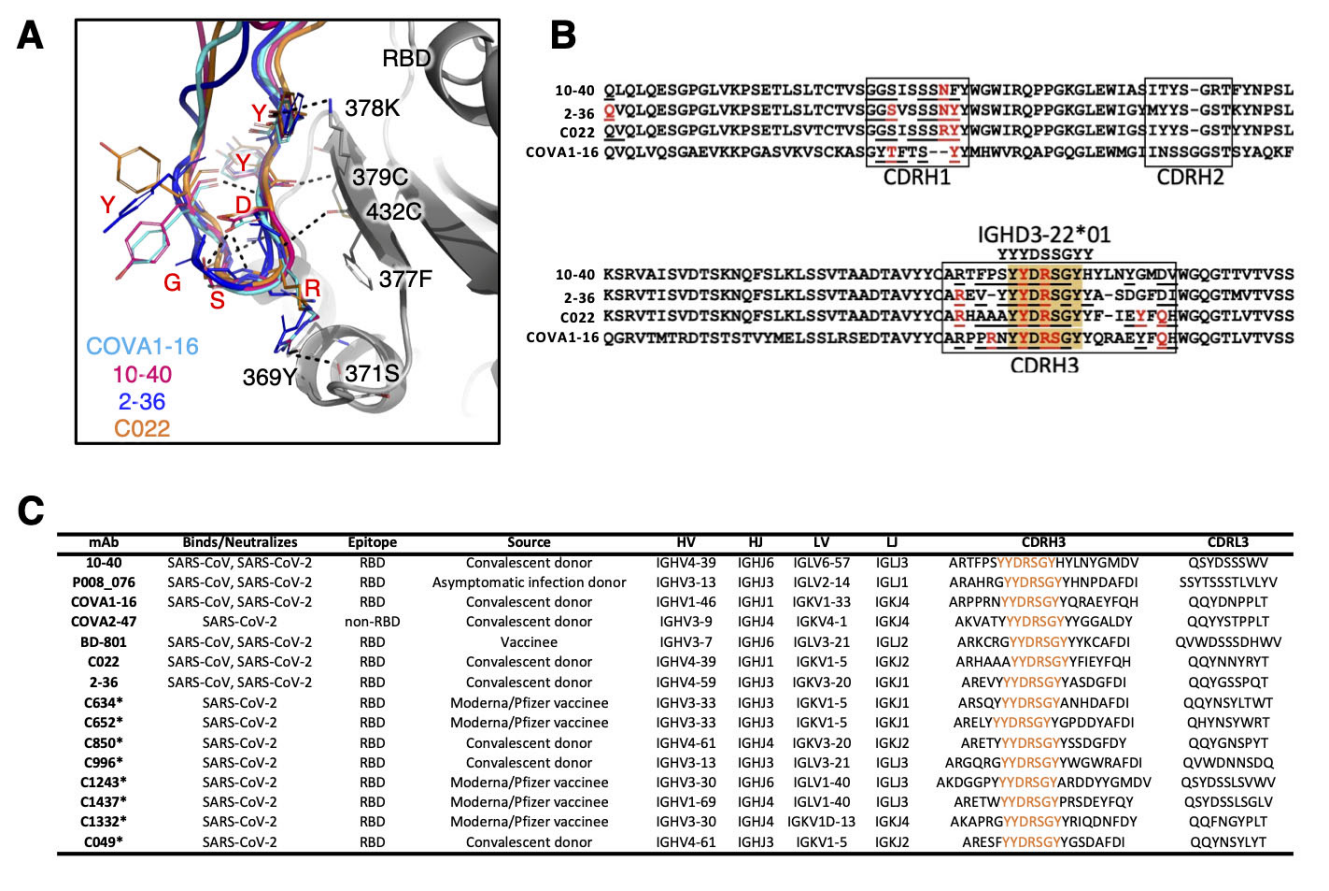
**The CDRH3 ‘YYDRSGY’ motif is conserved in 10-40-like broadly neutralizing antibodies. (A)** CDRH3s from 10-40, COVA1-16, and 2-36 are shown by superimposing RBDs from each complex, revealing a similar binding mode. The RBD is shown in gray. **(B)** Heavy chain sequence alignment is shown for 10-40, 2-36, C022, and COVA1-16, with the CDRH3 aligned with the germline sequence of the IGHD3-22*01 gene. The paratope residues are underlined and residues that form hydrogen bonds with RBD are colored in red. The 'YYDRSGY’ motif is highlighted in orange. **(C)** Published SARS-CoV-2 neutralizing antibodies that include the ‘YYDRSGY’ motif are shown. The ‘YYDRSGY’ motif is highlighted in orange for each CDRH3. P008_076 is from (*56*), COVA1-16 and COVA-2-47 are from (*57*), BD-801 is from (*58*), C022 is from (*59*), 2-36 is from (*12*), C634 and C652 are from (*21*), C850 and C996 are from (*60*), C1243, C1437, and C1332 are from (*61*), and C049 is from (*62*).

Given the broad recognition of 10-40 for sarbecoviruses, we carefully analyzed the amino acids that form its epitope and noted their remarkable conservation among sarbecoviruses (**Fig. 6A** **, fig. S16**), suggesting that this RBD region was likely subjected to strong functional constraints during evolution of this subgenus. Residues 377-379 in a β-sheet was specifically targeted by the CDRH3 of 10-40 through multiple hydrogen bonds (**Fig. 6B**). COVA1-16, 2-36, and C022 interacted quite similarly with the same residues. We also noticed that these three mAbs, together with 10-40, contact this particular β-sheet through a ‘YYDRSGY’ motif originating from *IGHD3-22* (**Fig. 6B**), a D gene that is frequently used by antibodies in the human repertoire (**fig. S17**). Within publicly available SARS-CoV-2 antibody sequences, this motif was found in 15 antibodies, and was almost exclusively found in broad RBD-directed antibodies targeting this same epitope (**Fig. 6C**). This motif contains a Ser-to-Arg substitution, which formed hydrogen bonds with 369Y and 371S on RBD (**Fig. 6A**). We believe these structural similarities define 10-40, 2-36, C022, and COVA1-16 as members of a new antibody class, each using a shared mode of heavy chain binding to RBDs of sarbecoviruses (**Fig. 6C**). As these four mAbs use diverse heavy chain V genes and light-chain recombinations (**table S4**) and show a low degree of somatic hypermutation (**fig. S5**), the elicitation of this class of antibodies may not be overly difficult. This observation bodes well for the development of a pan-sarbecovirus vaccine.

### In vivo efficacy of 10-40

**Fig. 7.**
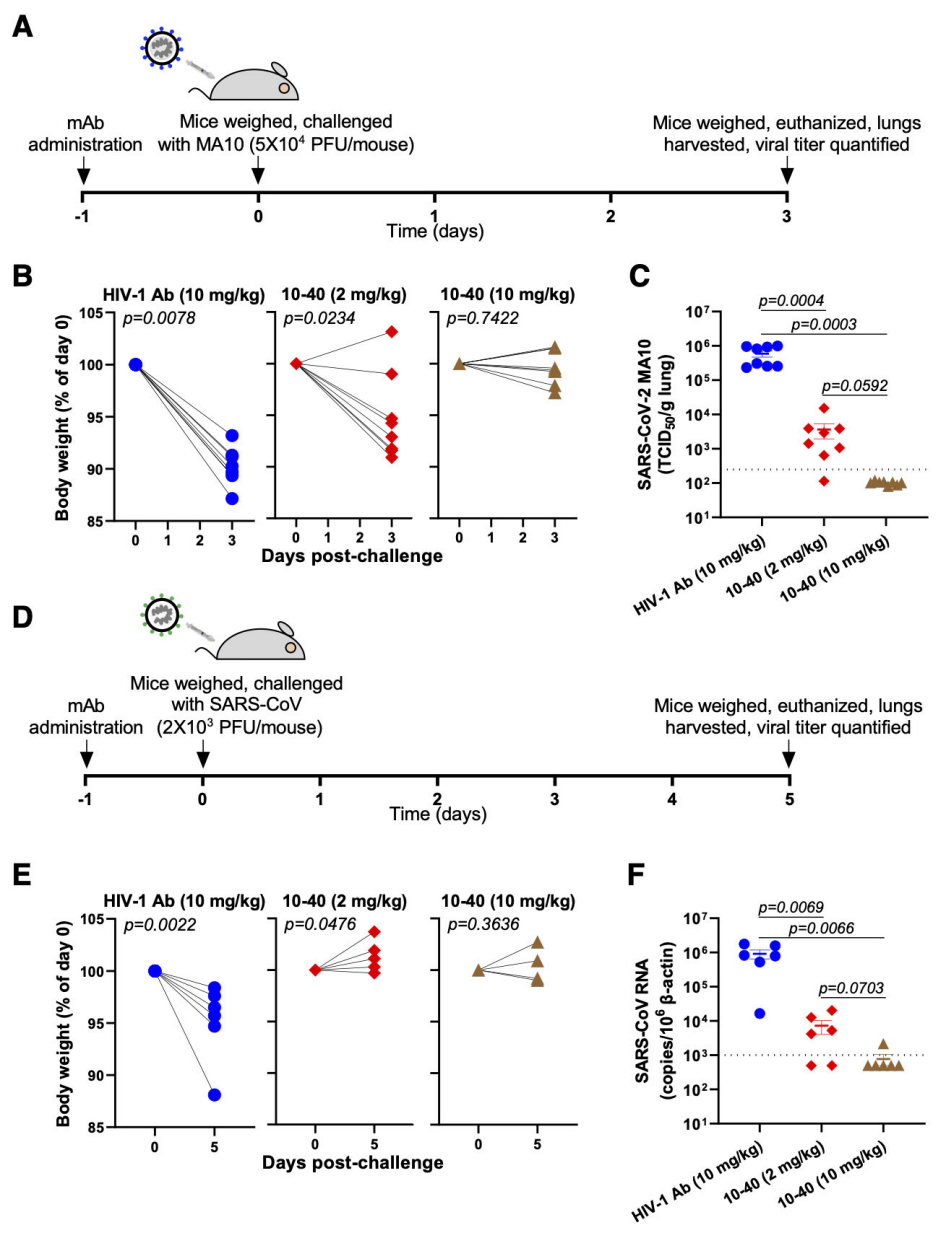
**Prophylactic protection against a mouse-adapted strain of SARS-CoV-2 (MA10) and SARS-CoV is conferred by 10-40. (A)** Experimental timeline of the protection study in MA10-challenged mice. PFU, plaque-forming units **(B)** Body weight change was measured for individual mice in each treatment group (n=8 mice per group). HIV-1 Ab was used as a control. P values were determined by two-tailed *t* test with Wilcoxon matched-pairing. **(C)** TCID_50_/g of lung was quantified for individual mice in each treatment group. Data are shown as mean ± SEM. The horizontal dotted line indicates the LOD of 250. **(D)** Experimental timeline of the protection study in SARS-CoV-challenged mice. **(E)** Body weight change was measured for individual mice in each treatment group (n=6 mice per group). HIV-1 Ab was used as a control. P values were determined by two-tailed *t* test with Wilcoxon matched-pairing. **(F)** SARS-CoV RNA (normalized to β-actin) was measured within lung from individual mice. Data are shown as mean ± SEM. The horizontal dotted line indicates the LOD of 1000.

Finally, we evaluated the in vivo protective efficacy of 10-40 by challenging wild-type mice with a mouse-adapted SARS-CoV-2 strain, MA10 (*24*), or by challenging K18-hACE2-transgenic mice with SARS-CoV (*25*). The RBD mutations in MA10 (Q493K, Q498Y, and P499T) mapped outside of the 10-40 epitope (**fig. S18A**) and did not strongly affect the neutralizing activity of 10-40 in vitro (**fig. S18B**). We then performed a prevention experiment (**Fig. 7A**), administering 10-40 or an anti-HIV-1 control mAb 24 hours before the mice were challenged intranasally with MA10. Compared to the control group, significant weight loss was prevented (10 mg/kg, p=0.7422) or mitigated (2 mg/kg, p=0.0234) by 10-40 administration (**Fig. 7B**). In mice given the control antibody, high titers of infectious virus were observed in the lungs (greater than 10^5^ median tissue culture infectious dose (TCID_50_)/g lung), whereas little (less than 10^4^ TCID50/g lung) or no infectious virus was found in mice given 10-40 at 2 mg/kg or 10 mg/kg, respectively (**Fig. 7C**). An analogous prevention experiment was conducted against SARS-CoV in hACE2-transgenic mice (**Fig. 7D**). Weight loss was again prevented by 10-40 administration (**Fig. 7E**), and titers of SARS-CoV were markedly reduced in the lungs of mice pre-treated with 10-40 (**Fig. 7F**). 10-40 appeared to be active in vivo against both SARS-CoV-2 and SARS-CoV.

## DISCUSSION

Sarbecoviruses have caused two major outbreaks in humans in the past two decades: SARS-CoV in 2002 to 2003 and SARS-CoV-2 now. Thus, the world must prepare for the possibility of a future epidemic or pandemic due to another member of this subgenus that is presently harbored by bats and other animals (*5*, *17*, *26*). Pan-sarbecovirus neutralizing mAbs and vaccines could be useful interventions to contain another outbreak. Here, we have identified a human mAb, 10-40, that suits this need. 10-40 neutralized or bound to every sarbecovirus we tested, regardless of their usage of ACE2 as receptor. It exhibited potency against SARS-CoV-2 in vitro and in vivo, and its potency against other sarbecoviruses was even better, despite being isolated from a patient with COVID-19. Very recently, three sarbecoviruses closest genetically to SARS-CoV-2 have been identified in bats in Laos (*26*), along with two more in a different sublineage. Although not empirically tested, we note that they are likely to be susceptible to 10-40 because the key amino acids that would form this RBD epitope are identical to those in either GD-Pangolin or RmYN02, both of which were neutralized or bound by 10-40. Therefore, we believe 10-40 is a promising candidate for pandemic preparedness. As SARS-CoV-2 continues to evolve, new variants may arise that elude current mAbs but remain susceptible to 10-40. More importantly, it is imperative that we are prepared for a future zoonotic spillover event that may well occur.

The pursuit of a pan-sarbecovirus vaccine is already underway, including strategies that specifically target the stem helix in the S2 region of the S protein (*27*) or conserved elements within the RBD. Efforts directed to the latter have already shown promise (*28*–*30*). Our comparison of the antibodies directed to the conserved epitope delineated in this study highlight the structural differences in the epitopes recognized by 10-40 and DH1047 versus epitopes recognized by mAbs (such as ADG-2 or S2X259) with lower breadth against sarbecoviruses, which could be informative in focusing the antibody response to certain conserved residues on the inner face of RBD. The epitopes of 10-40 and DH1047 are substantially overlapping and target the same exposed β-sheet, but their angles of approach are different, suggesting that this site could be attacked in more than one way. This angle may be critical as antibodies with similar affinities, such as 10-40, COVA1-16, and 2-36, have differing potencies, although this will need to be investigated. Moreover, by genetically comparing 10-40 with other antibodies, we have uncovered a unique class of mAbs that target this particular β-sheet in RBD through a common *CDRH3* motif. That this class of mAbs could use multiple heavy and light chain V genes and did not require extensive somatic hypermutation is certainly welcome news for the development of a pan-sarbecovirus vaccine targeting the RBD.

There are some limitations to this study to be considered when interpreting the results. Although we investigated many RBDs and S proteins for binding and neutralization studies, we could not exhaustively test all known sarbecoviruses. Furthermore, for the non-human ACE2 utilizing sarbecoviruses, we could not assay for neutralization, and only quantified antibody binding. For the identified *CDRH3* motif, we did not experimentally validate whether it could be successfully utilized in vaccination. Additional studies are warranted to further investigate these possibilities. Nevertheless, the identification of 10-40 as a potential pandemic preparedness agent, and the comparative analyses presented herein, could help to guide future anti-coronavirus efforts.

## MATERIALS AND METHODS

### Study design

The objective of this study was to identify broadly neutralizing antibodies against sarbecoviruses, and then to conduct a thorough comparative analysis between these antibodies with those previously known. To do so, we screened serum samples from convalescent COVID-19 patients for those with broad neutralizing capability, sorted for S protein-binding memory B cells from these individuals, and then expressed and tested the binding and neutralization capability of the identified antibodies. The three that were identified to have broad reactivity, 10-40, 10-28, and 11-11, were then interrogated in relation to other known antibodies, which we determined from a literature search. Binding and neutralization experiments were conducted in technical duplicate or triplicate, respectively, and were experimentally replicated three times, of which a representative result is shown. For the most promising antibody, 10-40, we proceeded to solve structures by cryo-EM and X-ray crystallography and validate its efficacy in vivo against both SARS-CoV-2 and SARS-CoV. The mice in the in vivo experiments were allocated randomly with sample sizes similar to those used in related studies (n=6 to 8 per group), which we have previously observed to produce replicable results. Experimenters were not blinded for the experiments in this study and no data were excluded.

### Sample collection

The protocols for acquisition of samples were reviewed and approved by the Institutional Review Boards of Columbia University (Protocol# IRB-AAAS9722) and Hackensack Meridian School of Medicine (Protocol# Pro2020-0633). Informed consent was obtained from both patients. Patient 10 was symptomatic for COVID-19 in March 2020 and blood collection was performed in April 2020. Patient 11 was symptomatic for COVID-19 in November 2020 followed by two doses of mRNA-1273 vaccine in January and February 2021, and blood collection was performed one week following the second vaccination. Sequencing confirmed that Patient 11 was infected with the R.1 variant of SARS-CoV-2.

### Expression and purification of S trimer and RBD proteins

The mammalian expression vector encoding the ectodomain of the SARS-CoV S trimer and the vectors encoding SARS-CoV and SARS-CoV-2 RBD fused to an HRV-3C protease cleavage site followed by a mFc tag and an 8 × His tag at the C terminus were kindly provided by Jason McLellan (*31*). The ectodomains with 2P and furin mutations of SARS-CoV-2 D614G and B.1.351 S trimers were synthesized, fused to an 8 × His tag at the C terminus and then cloned into the paH vector. Other sarbecovirus RBD-His expression vectors were kindly provided by Pamela J. Bjorkman (*28*). To purify the S trimer proteins and RBD proteins, each expression vector was transiently transfected into Expi293 cells using 1 mg/mL polyethylenimine (PEI, Polysciences). Five days after transfection, the SARS-CoV and SARS-CoV-2 RBDs were purified using protein A agarose (Thermo Fisher Scientific). In order to obtain untagged RBD of SARS-CoV and SARS-CoV-2, the mFc and 8 × His tags at the C terminus were removed by HRV-3C protease (Millipore-Sigma) and then purified using Ni-NTA resin (Invitrogen) followed by protein A agarose. The S trimers and other sarbecovirus RBDs were purified using Ni-NTA resin (Invitrogen).

### Sorting for B.1.351 S trimer-specific memory B cells and single-cell B cell receptor sequencing

Peripheral blood mononuclear cells from Patient 10, Patient 11, and one healthy donor were stained with LIVE/DEAD Fixable Yellow Dead Cell Stain Kit (Invitrogen) at ambient temperature for 20 min, followed by washing with RPMI-1640 complete medium (RPMI-1640 + 10% fetal bovine serum (FBS) + 100 U/mL penicillin/streptomycin (P/S)) and incubation with 10 μg/mL B.1.351 S trimer at 4°C for 45 min. Afterwards, the cells were washed again and incubated with a cocktail of flow cytometry and Hashtag antibodies, consisting of CD3 phycoerythrin (PE)-CF594 (BD Biosciences, Catalog #562406, RRID: AB_11154406), CD19 PE-Cy7 (BioLegend, Catalog #302216, RRID: AB_314246), CD20 allophycocyanin (APC)-Cy7 (BioLegend, Catalog #302314, RRID: AB_314262), IgM V450 (BD Biosciences, Catalog #561286, RRID: AB_10611713), CD27 peridinin chlorophyll protein (PerCP)-Cy5.5 (BD Biosciences, Catalog #560612, RRID: AB_1727457), anti-His PE (BioLegend, Catalog #362603, RRID: AB_2563634), and human Hashtag 3 (BioLegend, Catalog #394665, RRID: AB_2801033) at 4°C for 1 hour. Stained cells were then washed, resuspended in RPMI-1640 complete medium and sorted for B.1.351 S trimer-specific memory B cells (CD3^−^CD19^+^CD27^+^S trimer^+^ live single lymphocytes) by flow cytometry. The sorted cells were mixed with spike-in mononuclear cells, labeled with Hashtag 5 (BioLegend, Catalog #394669, RRID: AB_2801035) for Patient 10 and Hashtag 2 (BioLegend, Catalog #394663, RRID: AB_2801032) for Patient 11 and loaded into a 10X Chromium chip of the 5′ Single Cell Immune Profiling Assay (10X Genomics) at the Columbia University Single-Cell Analysis Core. Library preparation and quality control were performed according to the manufacturer’s protocol and sequenced on a NextSeq 500 sequencer (Illumina).

### Identification of S trimer-specific antibody transcripts

We used an analysis protocol similar to our previous study to identify S protein-specific antibody transcripts (*19*). Briefly, full-length antibody transcripts were assembled using the Cell Ranger V(D)J pipeline (version 3.1.0, 10X Genomics) with default parameters and the GRCh38 V(D)J germline sequence version 2.0.0 as reference. To distinguish cells from the antigen sort and spike-in, we first used the count module in Cell Ranger to calculate copies of all hashtags in each cell from the Illumina next generation sequencing raw reads. High-confidence antigen-specific cells were identified as follows. A cell must contain more than 100 copies of the antigen sort-specific hashtag to qualify as an antigen-specific cell. Because hashtags can fall off cells and bind to cells from a different population in the sample mixture, each cell usually has both sorted and spiked-in-specific hashtags. To enrich for true antigen-specific cells, the copy number of the specific hashtag has to be at least 1.5× higher than that of the non-specific hashtag. Low-quality cells were identified and removed using the cell-calling algorithm in Cell Ranger. Cells that did not have productive heavy and light chain pairs were excluded. If a cell contained more than two heavy or light chain transcripts, the transcripts with fewer than three unique molecular identifiers were removed. Cells with identical heavy and light chain sequences, which may be from mRNA contamination, were merged into one cell. We also applied additional filters to remove low-quality cells and transcripts in the antibody gene annotation process.

### Antibody transcript annotation

Antigen-specific antibody transcripts were processed using our bioinformatics pipeline SONAR version 2.0 for quality control and annotation (*32*). In brief, V(D)J genes were assigned for each transcript using BLASTn with customized parameters against a germline gene database obtained from the international ImMunoGeneTics information system (IMGT) database (*33*). On the basis of BLAST alignments of V and J regions, CDR3 was identified using the conserved second cysteine in the V region and WGXG (heavy chain) or FGXG (light chain) motifs in the J region (X represents any amino acid). For heavy chain transcripts, the constant domain 1 (CH1) sequences were used to assign isotype using BLASTn with default parameters against a database of human CH1 genes obtained from IMGT. A BLAST E-value threshold of 10^−6^ was used to find significant isotype assignments, and the CH1 allele with the lowest E-value was used. Sequences other than the V(D)J region were removed and transcripts containing incomplete V(D)J regions or frame shifts were excluded. We then aligned each of the remaining transcripts to the assigned germline V gene using CLUSTALO and calculated the degree of somatic hypermutation (*34*). The D gene assignment for each transcript was performed by the HighV-QUEST function in IMGT web server with the default parameters. For cells having multiple high quality heavy or light chains, which may be from doublets, we synthesized all H and L chain combinations.

### Antibody expression and purification

For each antibody, variable genes were optimized for human cell expression and synthesized by GenScript. VH and VL were inserted separately into pcDNA3.4 plasmids encoding the constant region for heavy chain and light chain, respectively. Monoclonal antibodies were expressed in Expi293F cells (Thermo Fisher Scientific, Catalog # A14527, RRID: CVCL_D615) by co-transfection of heavy chain and light chain expressing plasmids using PEI (Polysciences) and cultured in a 37°C shaker at 125 RPM under 8% CO_2_. Supernatants were collected five days post-transfection, and then antibodies were purified by affinity chromatography using rProtein A Sepharose (GE).

### Antigen binding testing by ELISA

Fifty ng per well of antigen (S trimer or RBD protein) was coated onto ELISA plates at 4°C overnight. The ELISA plates were then blocked with 300 μL blocking buffer consisting of phosphate-buffered saline (PBS) with 1% bovine serum albumin (BSA) and 10% bovine calf serum (BCS, Sigma Aldrich) at 37°C for 2 hours. Afterwards, 100 μL of serially diluted antibodies were added and then incubated at 37°C for 1 hour. Next, 100 μL of 10,000-fold diluted Peroxidase AffiniPure goat anti-human IgG Fcγ fragment specific antibody (Jackson ImmunoResearch, Catalog #109-035-170, RRID: AB_2810887) was added into each well and incubated for another 1 hour at 37°C. The plates were washed between each step with PBS-T (0.5% Tween-20 in PBS). Finally, 3,3′,5,5′-tetramethylbenzidine (TMB) substrate (Sigma Aldrich) was added and incubated before the reaction was stopped using 1 M sulfuric acid. Absorbance was measured at 450 nm.

### Antigen binding testing by SPR

SPR binding assays for IgG binding to S protein were performed using a Biacore T200 biosensor, equipped with a Series S CM5 chip (Cytiva), in a running buffer of 10 mM HEPES pH 7.4, 150 mM NaCl, 3 mM EDTA, 0.05% P-20 (HBS-EP+ buffer, Cytiva) at 25°C. SARS-CoV-2 or SARS-CoV S protein was captured through its C-terminal His-tag over an anti-His antibody surface. These surfaces were generated using the His-capture kit (Cytiva) according to the manufacturer’s instructions, resulting in approximately 10,000 resonance units (RU) of anti-His antibody over each surface. S protein was captured over a single flow cell at 125 to 200 RU. An anti-His antibody surface was used as a reference flow cell to remove bulk shift changes from the binding signal. IgG antibodies were tested using a three-fold dilution series of IgG antibodies with concentrations ranging from 1.2 nM to 33.3 nM. The association and dissociation rates were each monitored for 55 s and 300 s respectively, at 50 mL/minute. The bound S protein-IgG complexes were regenerated from the anti-His antibody surface using 10 mM glycine pH 1.5. Blank buffer cycles were performed by injecting running buffer instead of IgG to remove systematic noise from the binding signal. The resulting data was processed and fitted to a 1:1 binding model using Biacore Evaluation Software.

### ACE2 competition and epitope mapping by ELISA

For the competition ELISA, purified antibodies and ACE2 protein (Sino Biological) were biotin-labeled using One-Step Antibody Biotinylation Kit (Miltenyi Biotec) following the manufacturer’s instructions and purified using 40K MWCO Desalting Column (Thermo Fisher Scientific). Serially diluted competitor antibodies (50 μL) were added into S trimer-precoated ELISA plates, followed by 50 μL of biotinylated antibodies at a concentration that achieved an OD450 reading of 2.5 in the absence of competitor antibodies. Plates were incubated at 37°C for 1 hour, and then 100 μL of 500-fold diluted Avidin-horseradish peroxidase (HRP, Thermo Fisher Scientific) was added into each well and incubated for another 1 hour at 37°C. The plates were washed with PBST between each of the steps. The plates were developed afterwards with TMB and absorbance was read at 450 nm after the reaction was stopped with 1 M sulfuric acid. For all the competition ELISA experiments, the relative binding of biotinylated antibodies and ACE2 to the S trimer in the presence of competitors was normalized by comparing to competitor-free controls. Relative binding curve and the area under curve (AUC) were determined in GraphPad Prism 9.2.

### S protein constructs for cell surface expression and production of pseudoviruses

The original SARS-CoV-2 S protein expression vector was purchased from Sino Biological (GenBank accession no. YP_009724390). This construct was modified as appropriate by site-directed mutagenesis to prepare plasmids encoding SARS-CoV-2 variant S proteins. SARS-CoV (GenBank accession no. AAP13567) and MERS-CoV (GenBank accession no. AFS88936) S protein expression vectors were also purchased from Sino Biological. Sarbecovirus S genes were codon optimized for mammalian expression, synthesized by Twist Biosciences, and cloned into the same expression vectors as above by Gibson Assembly (New England Biolabs). Sequences were retrieved from GenBank under the following accession numbers: GD-Pangolin (MT799524), GX-Pangolin (MT040333), RaTG13 (QHR63300), WIV1 (KF367457), SHC014 (KC881005), LYRa11 (KF569996), Rs7327 (KY417151), Rs4231 (KY417146), Rs4084 (KY417144), ZC45 (MG772933), Yunnan2011 (JX993988), As6526 (KY417142), Rs4237 (KY417147), Rs4081 (KY417143). The RmYN02 sequence was retrieved from Global Initiative on Sharing Avian Influenza Data (GISAID) (*35*) under the following accession number: EPI_ISL_412977.

### Cell surface S protein binding by flow cytometry

Expi293 cells were co-transfected with vectors encoding pRRL-cPPT-PGK-GFP (Addgene) and full-length S trimer of SARS-CoV, SARS-CoV-2, or other sarbecovirus at a ratio of 1 to 1. Three days after transfection, cells were incubated with 10 μg/mL antibody at 4°C for 1 hour. Then, 100 μL of APC anti-human IgG Fc (BioLegend, Catalog #366906, RRID: AB_2888847) at 1:20 dilution was added to the cells and incubated at 4°C for 45 min. Cells were washed 3 times with FACS buffer (PBS + 2% FBS) before each step. Lastly, cells were resuspended and antibody binding to cell surface S trimer was quantified on a LSRII flow cytometer (BD Biosciences). The mean fluorescence intensity (MFI) of antibody-bound APC-positive cells within green fluorescent protein (GFP)-positive cells were analyzed using FlowJo (BD Biosciences).

### Production of pseudoviruses

Recombinant vesicular stomatitis virus (rVSV) pseudoviruses in which the native glycoprotein was replaced with SARS-CoV S protein, S protein from SARS-CoV-2 or one of its variants, or other sarbecovirus S proteins were generated as previously described (*36*). HEK293T cells (American Type Culture Collection (ATCC), Catalog #CRL-3216, RRID: CVCL_0063) at a confluency of 80% were transfected with a S protein expression vector using 1 mg/mL PEI and cultured overnight at 37°C under 5% CO_2_. 24 hours later, cells were infected with VSV-G pseudotyped ΔG-luciferase (G*ΔG-luciferase, Kerafast) at a multiplicity of infection (MOI) of 3 for 2 hours. Afterwards, cells were washed three times with 1× PBS, changed to fresh medium, and cultured at 37°C for another 24 hours before supernatants were harvested and clarified by centrifugation at 300 × g for 10 min.

### Pseudovirus neutralization assay

Pseudoviruses were first titrated to equilibrate viral input between assays. Neutralization assays were then performed by incubating pseudoviruses with serially diluted heat-inactivated serum or antibodies in triplicate in 96-well plate for 1 hour at 37°C. For neutralization of SARS-CoV and SARS-CoV-2 pseudoviruses, Vero-E6 cells (ATCC, Catalog #CRL-1586, RRID: CVCL_0574) were seeded at a density of 4 × 10^4^ cells per well, whereas for neutralization of pseudoviruses derived from other sarbecoviruses, 293T-hACE2 (*36*) were seeded at a density of 1 × 10^5^ cells per well. Then, luciferase activity was measured using the Luciferase Assay System (Promega), according to the manufacturer’s instructions, 24 hours after cells were added to the pseudovirus and antibody or serum mixture. The neutralization curves and IC_50_ values were generated by fitting a nonlinear five-parameter dose-response curve in GraphPad Prism 9.2.

### Authentic virus propagation and titration

The SARS-CoV-2 viruses USA-WA1/2020 (WA1), USA/CA_CDC_5574/2020 (Alpha variant, B.1.1.7), hCoV-19/South Africa/KRISP-EC-K005321/2020 (Beta variant, B.1.351), hCoV-19/Japan/TY7-503/2021 (Gamma variant, P.1), hCov-19/USA/NY-MSHSPSP-PV29995/2021 (Delta Variant, B.1.617.2) and SARS-CoV-2, mouse adapted MA10, infectious clone (USA-WA1/2020 background, (*24*)) were obtained from Biodefense and Emerging Infections Research Resources Repository (BEI Resources) at the National Institute of Allergy and Infectious diseases (NIAID) within the National Institutes of Health (NIH) and propagated for one passage using Vero-E6 cells. Virus infectious titer was determined by an end-point dilution and cytopathic effect (CPE) assay on Vero-E6 cells as described previously (*19*).

### Neutralization of authentic SARS-CoV-2 by purified monoclonal antibodies

An end-point dilution assay in a 96-well plate format was performed to measure the neutralization of SARS-CoV-2. In brief, purified monoclonal antibodies were serially diluted at 5-fold dilutions starting at 50 μg/mL. Triplicates of each dilution of the antibody were incubated with SARS-CoV-2 at an MOI of 0.1 in Eagle's Minimum Essential Medium (EMEM, ATCC) with 7.5% inactivated FBS for 1 hour at 37°C. Post incubation, the virus-antibody mix was transferred onto a monolayer of Vero-E6 cells grown overnight. The cells were incubated with the mixture for 70 hours. Morphological changes resulting from CPE due to infection of cells were visually scored for each well from 0 to 4 with 4 defined as the appearance of complete virus mediated cytopathy. Double-blinded scoring of the cytopathic effect was converted to percentage of neutralization, and the IC_50_ was determined by fitting a five-parameter dose-response curve using GraphPad Prism v9.2.

### Neutralization of authentic SARS-CoV by purified monoclonal antibodies

To measure neutralization of authentic SARS-CoV, Vero-E6 cells were seeded in 96 well-plates in cell culture media (Dulbecco's Modified Eagle Medium (DMEM) + 10% FBS + 1% P/S) overnight at 37°C under 5% CO_2_ to establish a monolayer. The next day, antibodies were diluted as appropriate in DMEM + 2% FBS and incubated with 0.01 MOI of SARS-CoV GZ50 strain (GenBank accession no. AY304495) for 1 hour at 37°C in quadruplicate (*37*). After incubation, the antibody-virus mixture was overlaid onto cells and incubated at 37°C under 5% CO_2_ for 72 hours. CPE was then visually scored as either negative or positive for infection by comparison to control uninfected or infected wells in a blinded manner by two independent observers. Each concentration of antibody was tested in triplicate, and the percentage of infected wells was used to calculate IC_50_ by fitting a five-parameter dose-response curve using GraphPad Prism v9.2.

### Protein expression for cryo-electron microscopy (cryo-EM) and crystallography

The RBD (residues 319 to 541) of the SARS-CoV-2 was expressed and purified as previously described (*38*). Briefly, protein expression was carried out in suspension cultures of Human Embryonic Kidney (HEK) 293 GnTI-Freestyle cells using serum-free media (Invitrogen) by transient transfection using PEI (Polysciences). Media was harvested four days after transfection and the secreted protein was purified from the supernatant using nickel affinity chromatography using Ni-NTA IMAC Sepharose 6 Fast Flow resin (GE Healthcare) followed by size exclusion chromatography (SEC) on a Superdex 200 column (GE Healthcare) in 20 mM Tris, 150 mM NaCl, pH 8.0.

Fab fragments of 10-40, 10-28, and 11-11 were produced by digestion of IgG antibodies with immobilized Endoproteinase Lys-C (Sigma Aldrich) equilibrated with 25 mM Tris pH 8.5 and 1 mM EDTA for 3 hours. The resulting Fabs were purified from the cleaved Fc domain by affinity chromatography using protein A. Fab purity was analyzed by SDS-PAGE. All Fabs were buffer-exchanged into 20 mM Tris, 150 mM, pH 7.4 prior to cryo-EM or crystallization experiments.

### Cryo-EM grid preparation

Samples for cryo-EM grid preparation were produced by mixing purified B.1.351 S protein (final protein concentration of 1 mg/mL) with Fabs in a 1:3 molar ratio, followed by incubation on ice for 1 hour. The final buffer for the S protein+Fab complex was 10 mM sodium acetate, 150 mM NaCl, pH 5.5. A final concentration of 0.005% (w/v) n-Dodecyl β-D-maltoside (DDM) was added to the mixtures to prevent preferred orientation and aggregation during vitrification. Cryo-EM grids were prepared by applying 3 μL of sample to a freshly glow-discharged carbon-coated copper grid (CF 1.2/1.3 300 mesh); the sample was vitrified in liquid ethane using a Vitrobot Mark IV with a wait time of 30 s, a blot time of 3 s, and a blot force of 0.

### Cryo-EM data collection and analysis

Cryo-EM data for single particle analysis was collected on a Titan Krios electron microscope operating at 300 kV, equipped with a Gatan K3-BioQuantum direct detection detector and energy filter, using the SerialEM software package (*39*). Exposures were taken with a total electron fluence of 41.93 e-/Å^2^ fractionated over 48 frames, with a frame time of 50 ms and a total exposure time of 2.4 s. A defocus range of -0.8 to -2.0 μm was used with a magnification of 81,000x, and a pixel size of 1.07 Å.

Data processing was performed using cryoSPARC v3.2.0 (*40*). Raw movies were aligned and dose-weighted using patch motion correction, and the contrast transfer function (CTF) was estimated using patch CTF estimation. Micrographs were picked using blob picker, and a particle set was selected using 2D and 3D classification. The resulting particle set was local motion corrected and refined to high resolution using a combination of heterogeneous and homogenous refinement. The final maps were deposited to the EMDB with ID: EMD-25146 (10-40), EMD-25166 (10-28), EMD-25167 (11-11).

### Footprint analysis

Initial molecular models for Fabs were generated using SAbPred server (*41*) and rigid body docked into the density map using Chimera “fit to map” tool (*42*). An RBD model (PDB 7BZ5) was also docked into the density map. The combined Fab+RBD model was saved and imported into PyMOL (version 2.5, Schrödinger, LLC). The Interface residues script was run using a dASA cutoff of 0.75 Å^2^. The resulting identified residues were colored by heavy and light chain and displayed as a molecular surface.

### Crystallization and data processing

10-40 + SARS-CoV-2-RBD, 10-28 + SARS-CoV-2-RBD, 10-40 + SHC014-RBD, 10-40 + RaTG13-RBD, and 10-40 + WIV1-RBD complexes were prepared by mixing each of the protein components at an equimolar concentration and incubating overnight at 4°C. Protein complexes were then isolated by gel filtration on a Superdex-200 column (GE Healthcare). Fractions containing complexes were pooled and concentrated to 8.0 mg/mL in SEC buffer (20 mM Tris pH 8.0, 150 mM NaCl). Screening for initial crystallization conditions was carried out in 96-well sitting drop plates using the vapor-diffusion method with a Mosquito crystallization robot (TTP LabTech) using various commercially available crystallization screens: JSCG+ (Qiagen), MSCG-1 (Anthracene) and LMB (Molecular dimensions). Diffraction quality crystals were obtained after 2 days in the following condition for each complex: 10-40 + SARS-CoV-2-RBD – 0.2 M Potassium thiocyanate, 0.1 M Sodium acetate pH 5.5, 8% w/v polyethylene glycol (PEG) 20,000, 8% v/v PEG 550 monomethyl ether (MME); 10-28 + SARS-CoV-2-RBD – 0.2 M (NH_4_)_2_SO_4_, 0.1 M HEPES pH 7.5, 25% w/v PEG 3350, 30% v/v PEG 2000MME; 10-40 + SHC014-RBD – 0.1 M Sodium citrate pH 5.2, 28% PEG 4000, 0.2 M NH_4_ acetate; 10-40 + RaTG13-RBD – 1 M LiCl, 0.1 M Sodium acetate, 30% PEG 6000; 10-40 + WIV1-RBD – 0.1 M Sodium citrate pH 5.5, 20% Propanol, 20% PEG 4000.

Prior to data collection, crystals were cryoprotected with 30% PEG 400 supplemented in mother liquor and flash frozen in liquid nitrogen. X-ray diffraction data extending to 1.40 Å (10-40 + SARS-CoV-2-RBD), 3.0 Å (10-28 + SARS-CoV-2-RBD), 2.2 Å (10-40 + SHC014-RBD), 2.8 Å (10-40 + RaTG13-RBD), and 3.1 Å (10-40 + WIV1-RBD) resolution were collected at 100 K on beamlines 24-ID-C and 24-ID-E of the Advanced Photon Source (APS). Diffraction data were processed with X-ray Detector Software (XDS) (*43*) and scaled using AIMLESS (*44*) from the CCP4 software suite (Collaborative Computational Project Number 4, 1994) (*45*). Molecular replacement was performed with Python-based Hierarchical ENvironment for Integrated Xtallography (PHASER) (*46*), using a previously reported SARS-CoV-2 RBD structure (PDB 7L5B) for 10-40 + SARS-CoV-2-RBD and 10-28 + SARS-CoV-2-RBD, a previously reported SARS-CoV-2 RBD structure (PDB 7SD5) with the corresponding residues mutated for 10-40 + SHC014-RBD and 10-40 + WIV1-RBD, a previously reported RaTG13 RBD structure (PDB 7DRV) for 10-40 + RaTG13-RBD and for 10-40 Fab, heavy chain (PDB 7RKU), light chain (PDB 4RIR) and for 10-28 Fab, heavy chain (PDB 6ZF0), light chain (PDB 70R9) used as search models. Manual rebuilding of the structure using Crystallographic Object-Oriented Toolkit (COOT) (*47*) was alternated with refinement using Phenix refine (*48*) and PDB-REDO (*49*). The Molprobity server was used for structure validation (*50*) and PyMOL (version 2.1, Schrödinger, LLC) for structure visualization. A summary of the X-ray data collection and refinement statistics are shown in **table S2** and **table S3**. Coordinates for the SARS-CoV-2 RBD in complex with 10-40 Fab, SARS-CoV-2 RBD in complex with 10-28 Fab, SHC014-RBD in complex with 10-40 Fab, RaTG13-RBD in complex with 10-40, and WIV1-RBD in complex with 10-40 were deposited to PDB under deposition codes 7SD5, 7SI2, 7TTM, 7TTX, and 7TTY, respectively. Epitope and paratope residues, as well as their interactions, were identified with Proteins, Interfaces, Structures and Assemblies (PISA) (*51*).

### Sequence conservation and phylogenetic analysis for sarbecoviruses

S protein sequences of 52 sarbecoviruses were obtained from NCBI with the accession numbers listed in **table S5**. The sequences were aligned with the MUltiple Sequence Comparison by Log-Expectation (MUSCLE) algorithm in MEGA7 with the default parameter (*52*, *53*). The conservation of each RBD residue was calculated using the entropy function of the R package bio3d (H.norm column) based on the RBD sequence alignment. Sequence entropy was visualized on the RBD structure using PyMol version 2.3.2. The maximum likelihood phylogenetic tree of sarbecoviruses was generated using MEGA7 by using the amino acid sequence alignment of RBDs with the general time reversible (GTR+G) substitution model, and the ancestor branch of BM4831 and BtY72 was used to root the phylogenic tree.

### Epitope and paratope analysis for antibody 10-40

The paratope and epitope residues for 10-40 were identified using PISA with the default parameters. 10-40 epitope residues from different sarbecoviruses were identified from the RBD sequence alignment described above. Positional frequency of amino acids within the 10-40 epitope was generated using WEBLOGO (*54*). The gene specific substitution profiles (GSSP) for 10-40 germline genes and IGHD3-22 gene usage in antibody repertoires were obtained from the curated antibody repertoires (cAb-Rep) database (*55*).

### Protection against mouse adapted SARS-CoV-2 challenge by 10-40

The mouse study was performed at the Columbia University Medical Center under a protocol (AC-AABK7562) approved by the Institutional Animal Care and Use Committee. All work was performed with approved standard operating procedures and the SARS-CoV-2 challenge was conducted in an animal biosafety level 3 (ABSL3) facility. 16-week-old female BALB/c mice (The Jackson Laboratory) were intraperitoneally injected with the indicated concentration of antibody 24 hours prior to infection. For infection, each mouse was anesthetized with a mixture of ketamine/xylazine and infected intranasally with 50 μL of PBS containing 5 × 10^4^ plaque-forming units (PFU) of mouse adapted SARS-CoV-2 MA10 strain (obtained from BEI Resources, NIAID, NIH, (*24*)) (n=8 per group). Mice were monitored for body weight changes at day 0 and day 3. At day 3 post-infection, mice were euthanized, and lung tissue was harvested and homogenized for viral titer analysis. Lung viral load titer was determined by an endpoint dilution assay.

### Protection against SARS-CoV challenge by 10-40

The in vivo anti-SARS-CoV activity of 10-40 was evaluated in the established hACE2-transgenic (human ACE2-transgenic) mouse model as described previously (*25*). These experiments were conducted under a protocol (5862-21) approved by the Hong Kong University (HKU) Committee on the Use of Live Animals in Teaching and Research (CULATR) and complied with the ARRIVE guidelines. Briefly, male hACE2-transgenic mice, aged 8 to 10 weeks, were obtained from the HKU Centre for Comparative Medicine Research and divided into different groups and intraperitoneally injected with the indicated concentration of antibody 24 hours prior to infection. The following day, mice were anesthetized with ketamine (100 mg/kg) and xylazine (10 mg/kg), and then intranasally inoculated with 20 μL of PBS containing 2000 PFU of SARS-CoV GZ50 strain (n=6 per group). Mice were monitored for body weight changes each day. The animals were euthanized at 5 dpi and lung tissue was harvested and homogenized for viral load quantitation by quantitative reverse transcription-polymerase chain reaction (RT-PCR) as previously described (*37*).

### Statistics

All raw, individual-level data are presented in data file S1. P values between groups were determined by two-tailed *t* test with Wilcoxon matched-pairing (**Fig. 7, B and E**) or two-tailed student’s unpaired *t* test (**Fig. 7, C and F**), using GraphPad Prism v9.2. All conducted statistical tests were two-tailed. P values of less than 0.05 were considered statistically significant. Data are presented as mean ± standard error of the mean (SEM) or standard deviation (SD) as described in the corresponding figure legends. Curve fitting was conducted by fitting a nonlinear five-parameter dose-response curve using GraphPad Prism v9.2.

## Supplementary Materials

### Other Supplementary Material for this manuscript includes the following:

MDAR Reproducibility Checklist
