# Supplementary material for: An antibody class with a common CDRH3 motif broadly neutralizes sarbecoviruses
Source: Sci Transl Med. 2022 Apr 19:eabn6859. doi: 10.1126/scitranslmed.abn6859 (PMC9017343; doi:10.1126/scitranslmed.abn6859)
Supplement: Supplementary file 1 — Figs. S1 to S18 Tables S1 to S5 [file scitranslmed.abn6859_sm.pdf]

Science Translational Medicine
AAAS

Supplementary Materials for

An antibody class with a common CDRH3 motif broadly neutralizes sarbecoviruses

Lihong Liu *et al.*

Corresponding authors: David D. Ho, dh2994@cumc.columbia.edu; Lawrence Shapiro, lss8@columbia.edu; Yaoxing Huang, yh3253@cumc.columbia.edu

DOI: 10.1126/scitranslmed.abn6859

The PDF file includes:

Figs. S1 to S18
Tables S1 to S5

Other Supplementary Material for this manuscript includes the following:

MDAR Reproducibility Checklist
Data file S1

Supplementary Figures

A

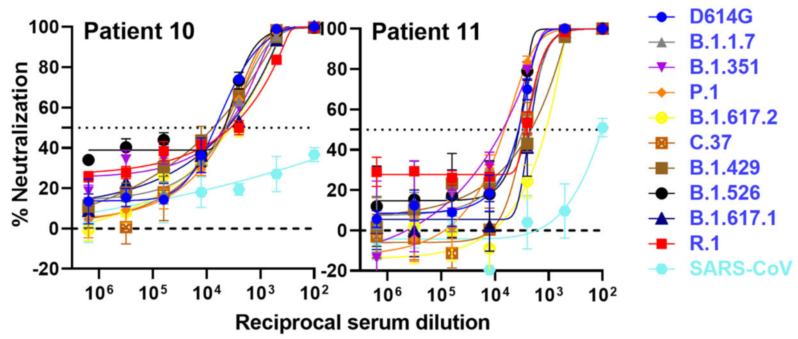

B

ID_{50}	Patient 10	Patient 11
D614G	6911	3635
B.1.1.7	4354	2967
B.1.351	4326	7147
P.1	4225	6625
B.1.617.2	3544	1126
C.37	4837	2781
B.1.429	7515	2112
B.1.526	4657	3562
B.1.617.1	4112	2238
R.1	3567	2641
SARS-CoV	>100	102

Fig. S1. Characterization of serum from two selected convalescent patients against pseudoviruses of SARS-CoV-2 variants or SARS-CoV. (A) Neutralization profiles and (B) 50% Infectious Dose (ID_{50}) titers are shown. SARS-CoV-2 variants are shown in blue, and SARS-CoV is shown in green. The dotted horizontal line in (A) indicates ID_{50} values. Data are shown as mean ± standard deviation (SD) of three technical replicates. SARS-CoV-2, severe acute respiratory syndrome coronavirus 2; SARS-CoV, severe acute respiratory syndrome coronavirus.

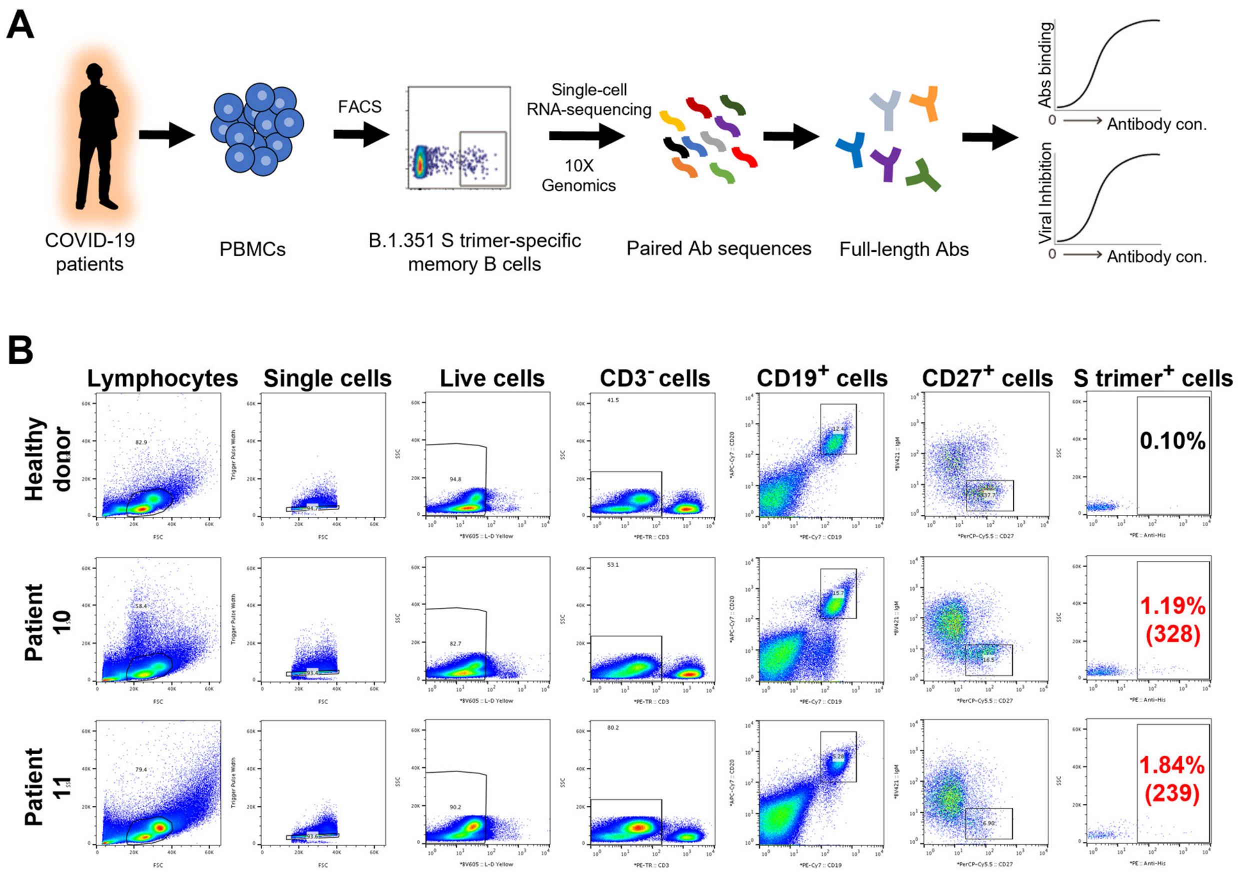

Fig. S2. Sorting strategy for B.1.351 S trimer-specific memory B cells. (A) Overview of the approach. PBMCs, peripheral blood mononuclear cells. Ab, antibody. **(B)** Gating scheme for sorting of trimer-specific memory B cells by flow cytometry. Inset numbers indicate the percentage (and the absolute number) of spike (S) trimer-specific memory B cells from each case.

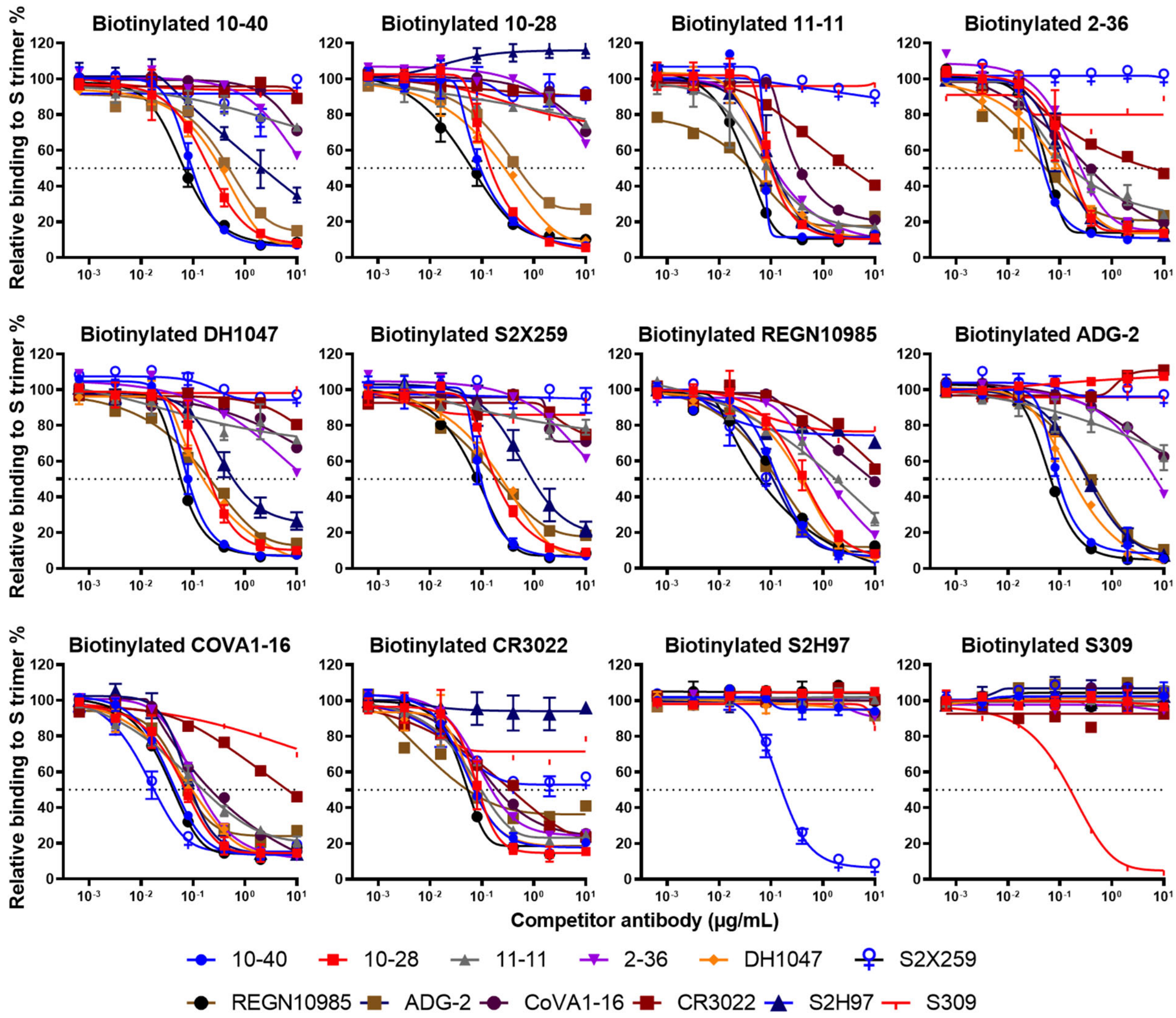

Fig. S3. Individual binding curves for competition enzyme-linked immunosorbent assay (ELISA) results summarized in Fig. 1C. Data are shown as mean ± SD of two technical replicates.

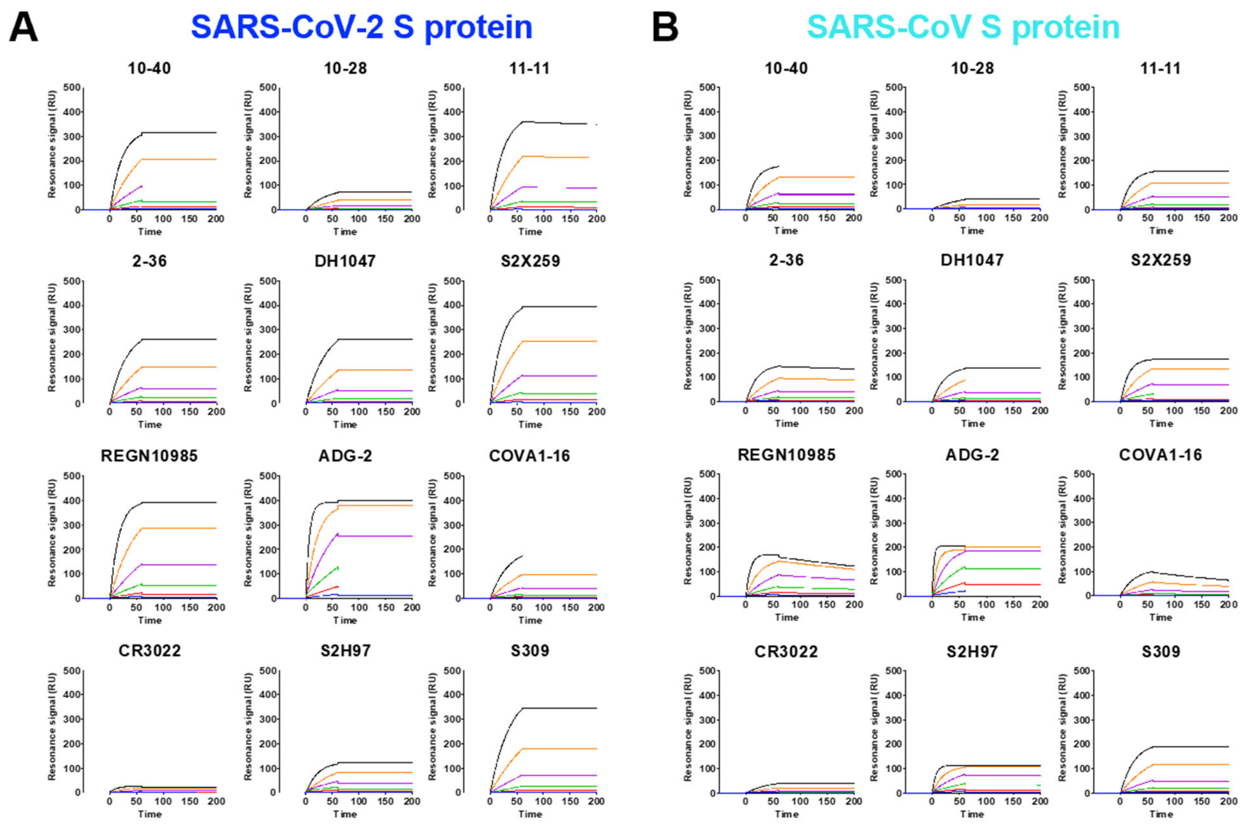

C

mAb	SARS-CoV-2 S protein			SARS-CoV S protein		
	k_a (1/Ms)	k_d (1/s)	K_D (M)	k_a (1/Ms)	k_d (1/s)	K_D (M)
**10-40**	1.60E+05	7.22E-07	4.52E-12	2.05E+05	4.54E-07	2.21E-12
**10-28**	9.17E+04	7.17E-08	7.82E-13	5.44E+04	2.01E-07	3.69E-12
**11-11**	1.36E+05	2.09E-04	1.54E-09	1.85E+05	8.34E-07	4.51E-12
**2-36**	1.11E+05	1.68E-06	1.51E-11	1.69E+05	4.99E-04	2.96E-09
**DH1047**	8.59E+04	9.10E-08	1.06E-12	1.52E+05	1.69E-07	1.11E-12
**S2X259**	1.54E+05	1.38E-07	8.95E-13	2.41E+05	8.78E-07	3.65E-12
**REGN10985**	2.10E+05	1.83E-06	8.72E-12	4.08E+05	1.90E-03	4.65E-09
**ADG-2**	5.09E+05	5.00E-07	9.83E-13	1.23E+06	1.73E-06	1.41E-12
**COVA1-16**	1.03E+05	1.33E-07	1.29E-12	1.64E+05	3.20E-03	1.95E-08
**CR3022**	2.71E+05	4.85E-04	1.79E-09	8.59E+04	6.52E-08	7.59E-13
**S2H97**	1.64E+05	7.42E-07	4.53E-12	5.20E+05	3.67E-05	7.06E-11
**S309**	9.18E+04	1.78E-07	1.94E-12	1.35E+05	2.63E-07	1.95E-12

Fig. S4. Binding kinetics and affinities of 10-40, 10-28, and 11-11, and other broadly neutralizing antibodies. Binding of antibodies to **(A)** SARS-CoV-2 and **(B)** SARS-CoV S proteins were determined by surface plasmon resonance (SPR). RU, resonance units. The S protein-bound sensors were incubated with six different concentrations of

antibodies. Kinetic data from one representative experiment were fitted to a 1:1 binding model. **(C)** Summary of SPR kinetic and affinity measurements.

A

mAbs	HV	HD	HJ	Isotype	H_CDR3_len	HCDR3	H_SHM(%)
**10-40**	*IGHV4-39*01*	*IGHD3-22*01*	*IGHJ6*02*	*IGHG1*04*	22	CARTFPSYYDRSGYHYLNYGMDVW	3
**10-28**	*IGHV3-30*18*	*IGHD2-2*03*	*IGHJ4*02*	*IGHG1*04*	13	CVKDGEQLVPLFDYW	2.4
**11-11**	*IGHV4-31*03*	*IGHD3-22*01*	*IGHJ6*02*	*IGHM*03*	21	CASSNYYDGSGYNLLESYGMDVW	0.7

mAbs	LV	LJ	L_CDR3_len	LCDR3	L_SHM(%)
**10-40**	*IGLV6-57*01*	*IGLJ3*02*	9	CQSYDSSSWVF	0.3
**10-28**	*IGKV1-39*01*	*IGKJ3*01*	10	CQQSYSTPGVTF	0.4
**11-11**	*IGLV1-40*01*	*IGLJ3*02*	12	CQSYDSSLSGSWVF	1

B

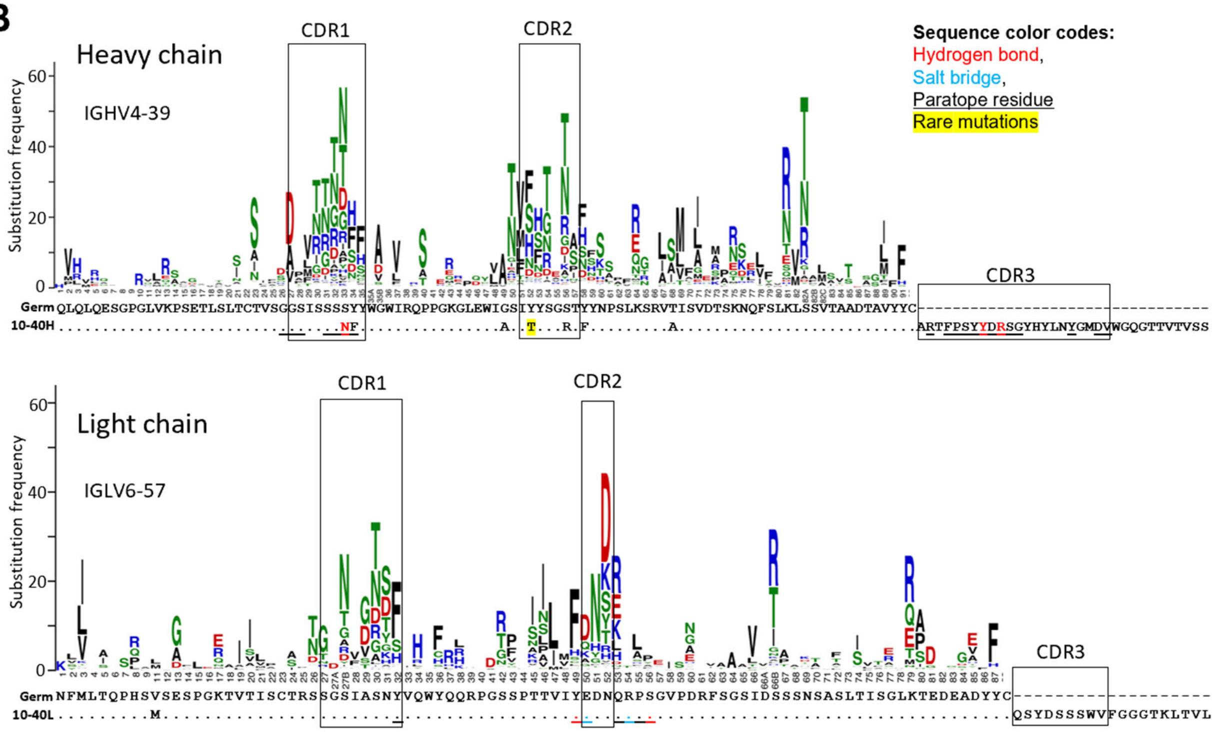

Fig. S5. Genetic analysis of 10-40, 10-28, and 11-11. (A) Genetic annotation is shown for the three broadly neutralizing antibodies identified in this study. HV, heavy variable ; HD, heavy diversity; HJ, heavy joining; HCDR3, heavy chain complementarity-determining region 3; H_SHM, heavy chain somatic hypermutation; LV, light variable; LJ, light joining; LCDR3, light chain complementarity-determining region 3; L_SHM, light chain somatic hypermutation. **(B)** Sequence alignment of 10-40 is shown with its germline genes, and gene-specific substitution profile (GSSP) for IGHV4-39 and IGLV6-57. The

dots denote conserved residues. Y52T in the 10-40 heavy chain is colored in yellow to indicate a rare mutation.

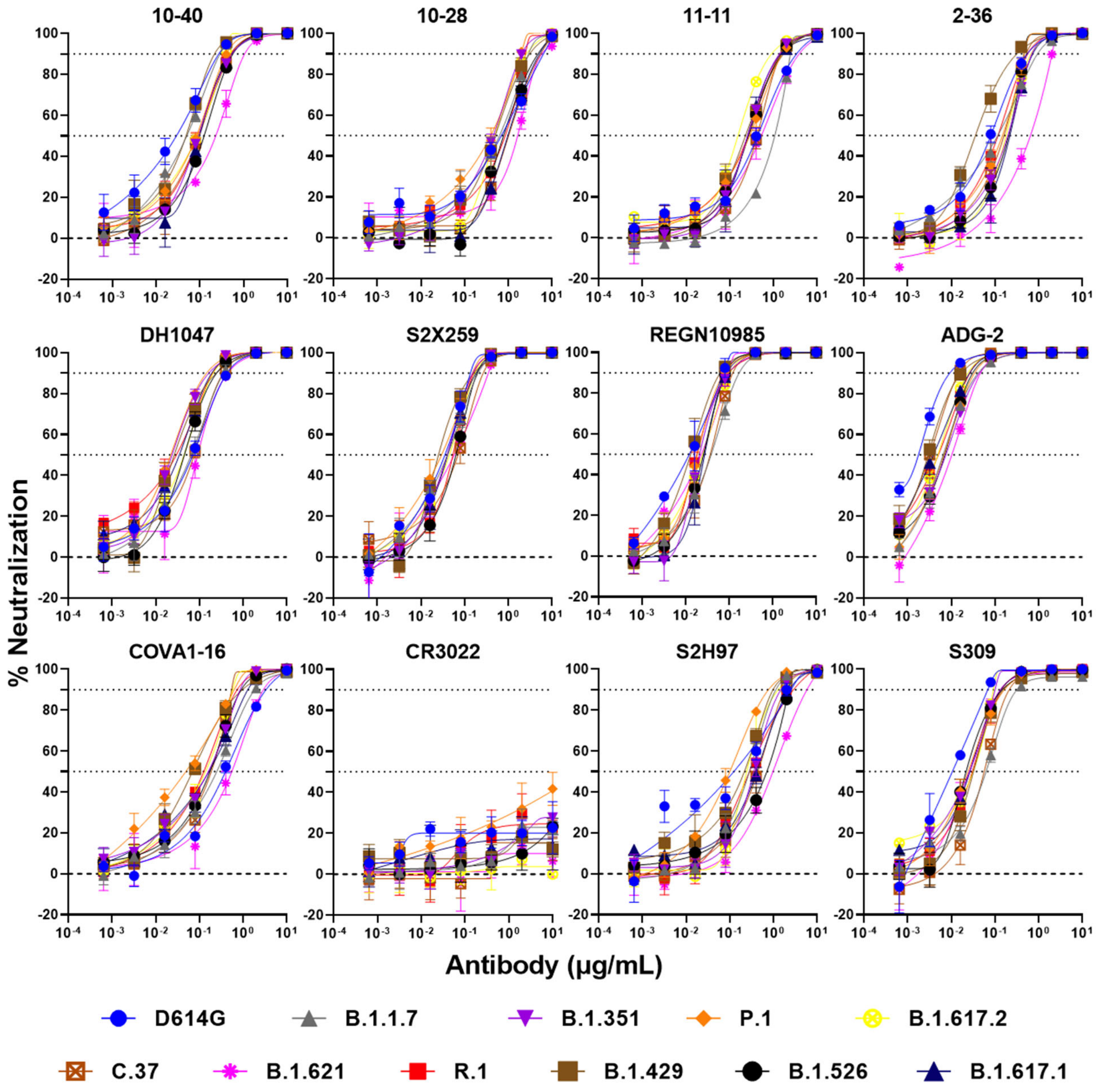

Fig. S6. Neutralization profiles of antibodies against SARS-CoV-2 variant pseudoviruses. Data are shown as mean ± SD of three technical replicates. Dotted lines represent 50% inhibitory concentration (IC_{50}) values and 90% inhibitory concentration (IC_{90}) values.

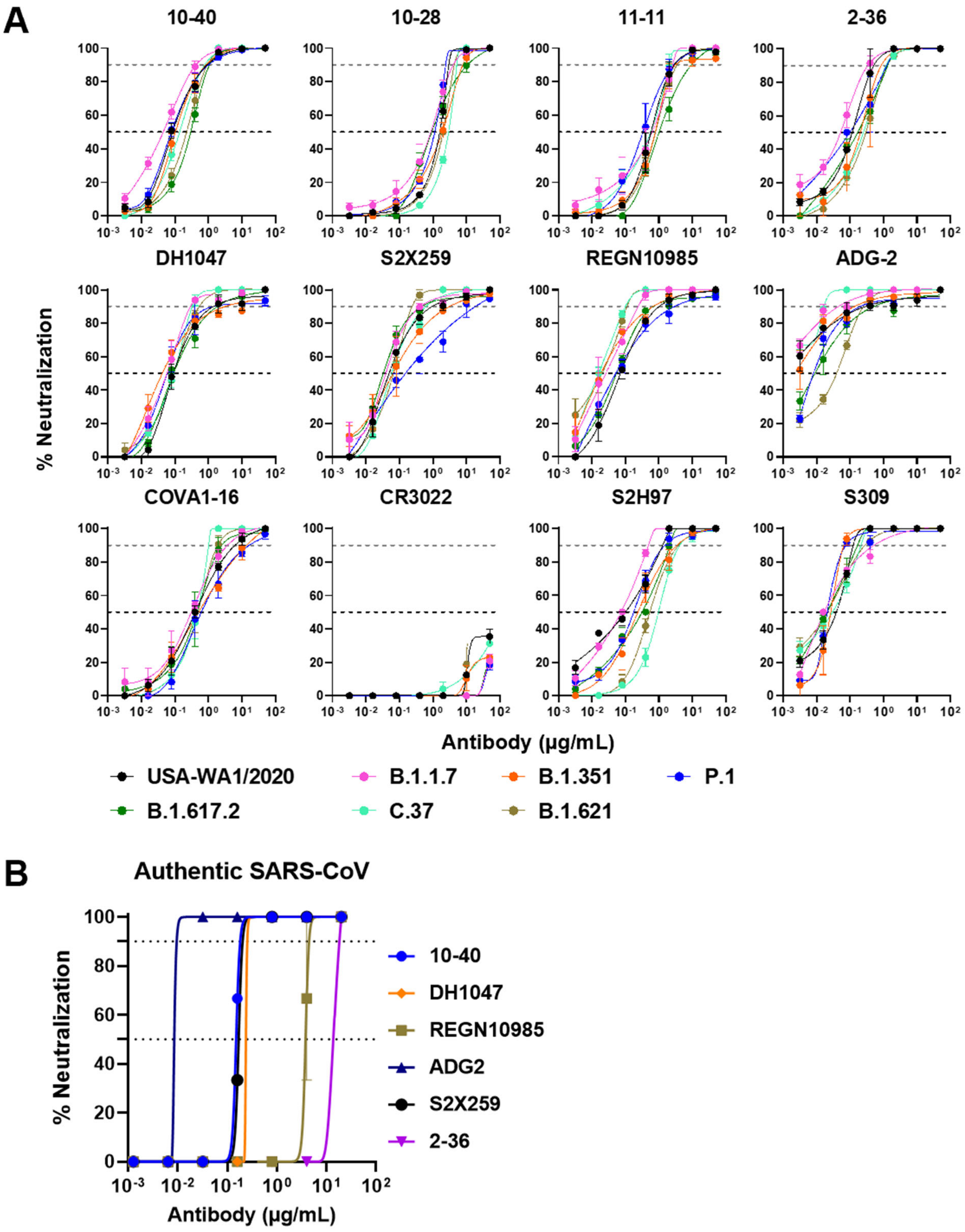

A
10-40
10-28
11-11
2-36
DH1047
S2X259
REGN10985
ADG-2
COVA1-16
CR3022
S2H97
S309
% Neutralization
Antibody (µg/mL)
USA-WA1/2020
B.1.1.7
B.1.351
P.1
B.1.617.2
C.37
B.1.621
B
Authentic SARS-CoV
% Neutralization
Antibody (µg/mL)
10-40
DH1047
REGN10985
ADG2
S2X259
2-36

Fig. S7. Neutralization profiles of antibodies against authentic viruses. Neutralization titers of **(A)** SARS-CoV-2 variants and **(B)** the SARS-CoV GZ50 strain are shown**.** Data are shown as mean ± standard error of the mean (SEM) of three technical replicates. Dotted lines represent IC_{50} and IC_{90} values.

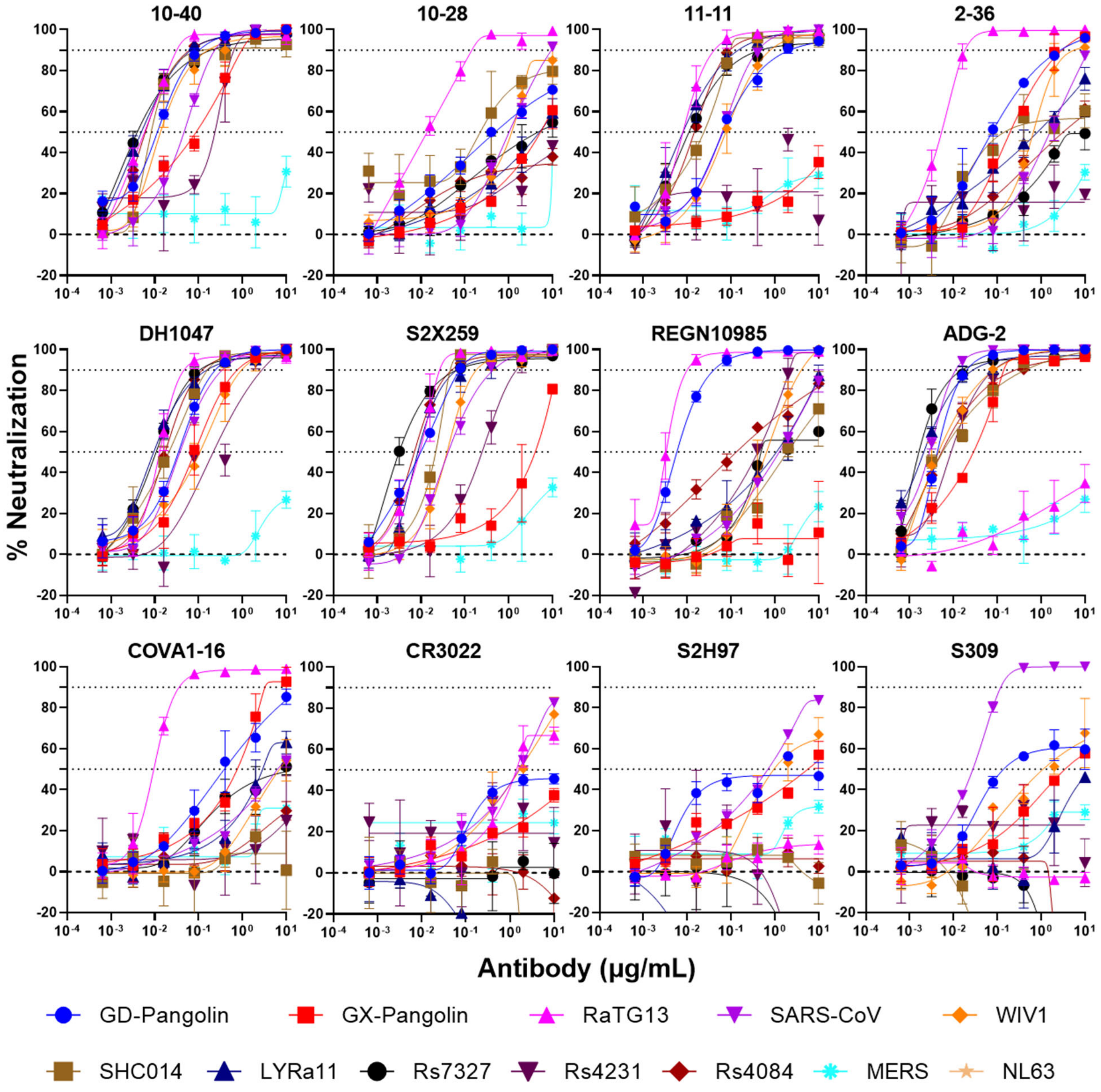

Fig. S8. Neutralization profiles of antibodies against sarbecoviruses. Data are shown as mean ± SD of three technical replicates. Dotted lines represent IC_{50} and IC_{90} values.

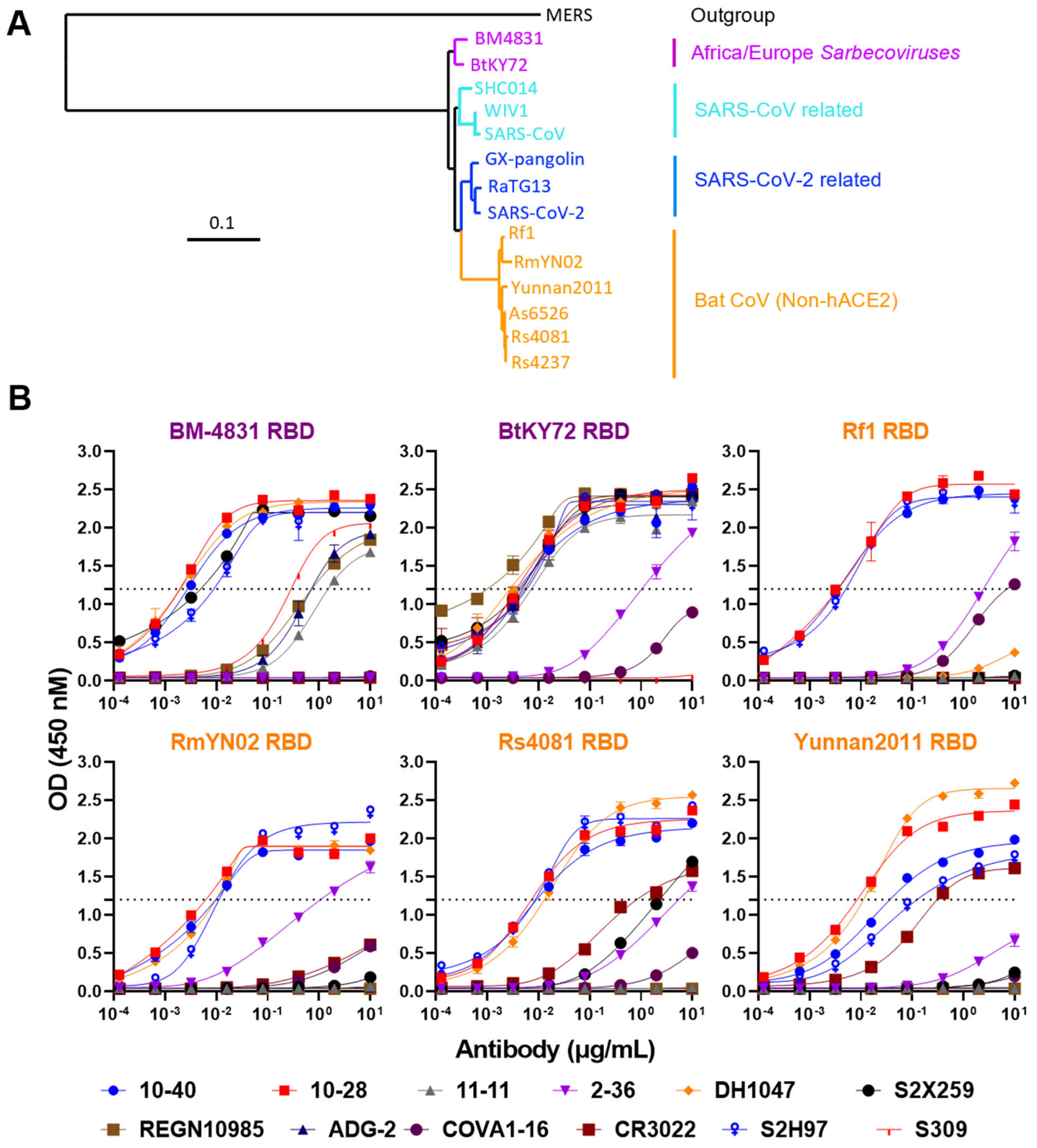

Fig. S9. Breadth of cross-reactive antibodies examined by receptor binding domain (RBD)-based ELISA. (A) A phylogenetic tree of sarbecoviruses is shown based on RBD protein sequences. Four phylogenetically distinct sarbecovirus groups are indicated in different colors. The scale bar indicates substitutions per site. **(B)** Binding of the

selected antibodies to the panel of RBD proteins as tested by ELISA. The dotted line indicates OD=1.2, the half-maximal binding value. OD, optical density.

Cell surface staining MFI	10-40	10-28	11-11	2-36	DH1047	S2X259	REGN 10985	ADG-2	COVA 1-16	CR3022	S2H97	S309
RmYN02	13124	14431	86	8349	11768	144	34	401	45	46	37	45
As6526	1633	1695	39	488	1786	41	33	38	31	29	32	105
Rs4237	1114	1131	93	415	1108	38	40	39	34	33	36	90

Fig. S10. Binding profiles of cross-reactive antibodies to non-human angiotensin-converting enzyme 2 (ACE2)-independent sarbecoviruses. Mean fluorescent intensities (MFI) are shown for antibodies bound to S protein-expressing cells. The data shown are representative data from one of three independent experiments.

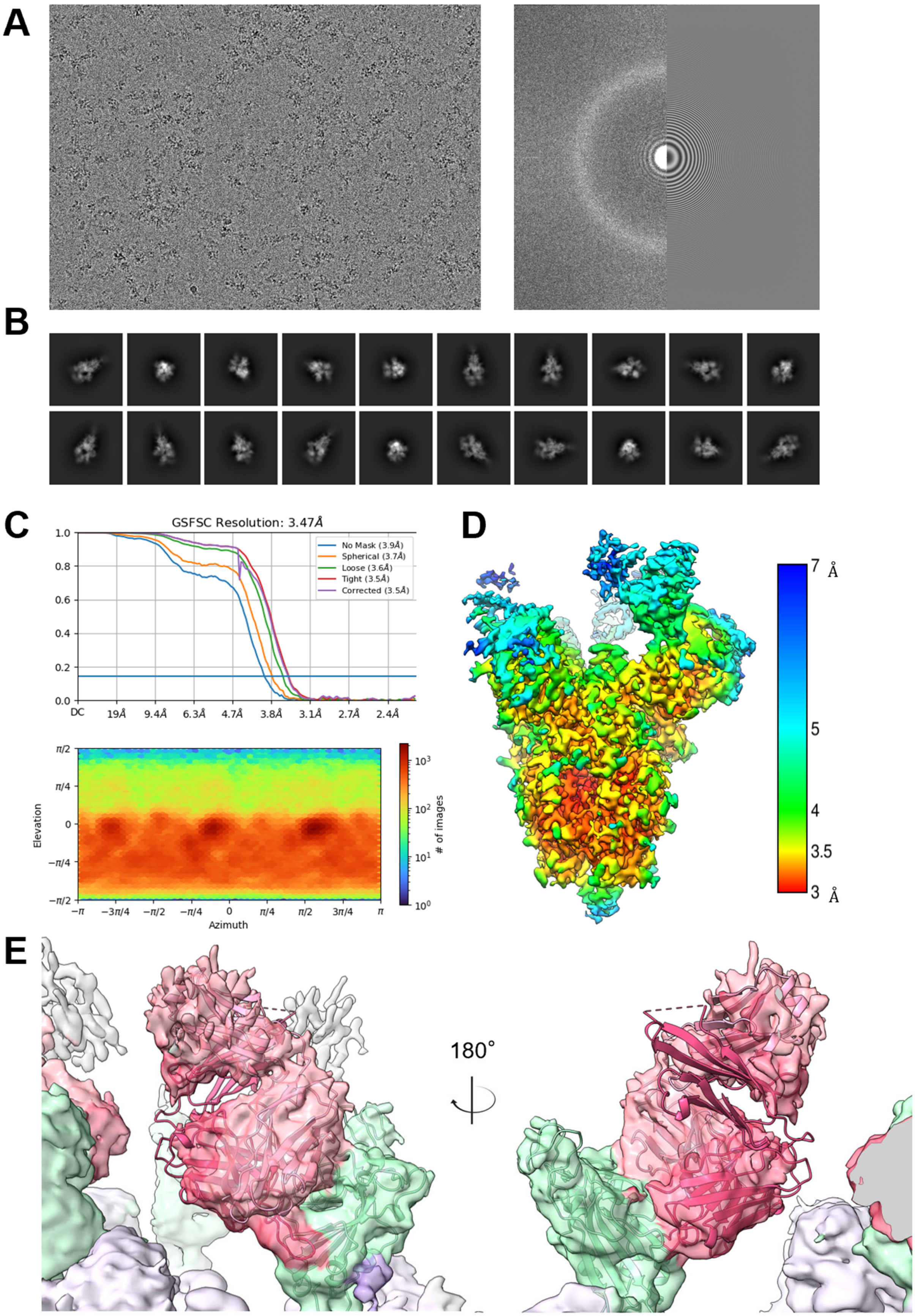

A
B
C
GSFSC Resolution: 3.47Å
No Mask (3.9Å)
Spherical (3.7Å)
Loose (3.6Å)
Tight (3.5Å)
Corrected (3.5Å)
DC
19Å
9.4Å
6.3Å
4.7Å
3.8Å
3.1Å
2.7Å
2.4Å
Elevation
Azimuth
# of images
D
7 Å
5
4
3.5
3 Å
E
180°

Fig. S11. Cryo-electron microscopy (EM) data processing for antibody 10-40 in complex with SARS-CoV-2 B.1.351 S trimer. (A) Representative micrograph, power spectrum, and contrast transfer function (CTF) fit. **(B)** Representative 2D class averages showing S particles. **(C)** Global consensus refinement Fourier Shell Correlation (FSC) curve (top panel) and particle projection viewing angle distribution (bottom panel). **(D)** Local resolution estimation mapped on surface density for global refinement. **(E)** Crystal structure model for 10-40 Fab rigid body docked into cryo-EM map, viewed from the outside (left) and from the inside (right). The Fab is shown in red and the RBD is shown in green.

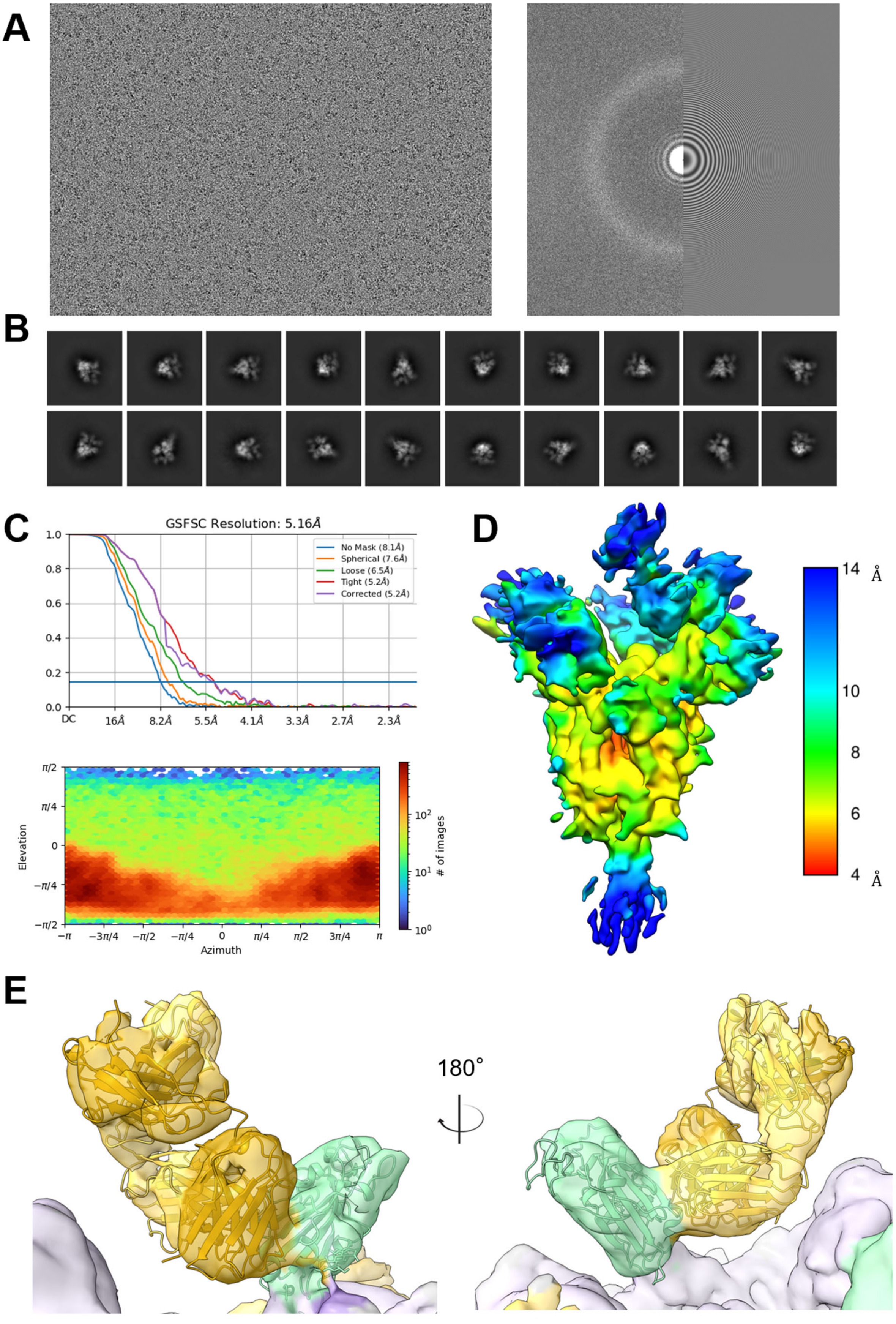

A
B
C
GSFSC Resolution: 5.16Å
No Mask (8.1Å)
Spherical (7.6Å)
Loose (6.5Å)
Tight (5.2Å)
Corrected (5.2Å)
1.0
0.8
0.6
0.4
0.2
0.0
DC
16Å
8.2Å
5.5Å
4.1Å
3.3Å
2.7Å
2.3Å
Elevation
Azimuth
# of images
D
14 Å
10
8
6
4 Å
E
180°

Fig. S12. Cryo-EM data processing for antibody 10-28 in complex with SARS-CoV-2 B.1.351 S trimer. (A) Representative micrograph, power spectrum, and CTF fit. **(B)** Representative 2D class averages showing S particles. **(C)** Global consensus refinement FSC curve (top panel) and particle projection viewing angle distribution (bottom panel). **(D)** Local resolution estimation mapped on surface density for global refinement. **(E)** Crystal structure model for 10-28 Fab rigid body docked into cryo-EM map, viewed from the outside (left) and from the inside (right). The Fab is shown in yellow and the RBD is shown in green.

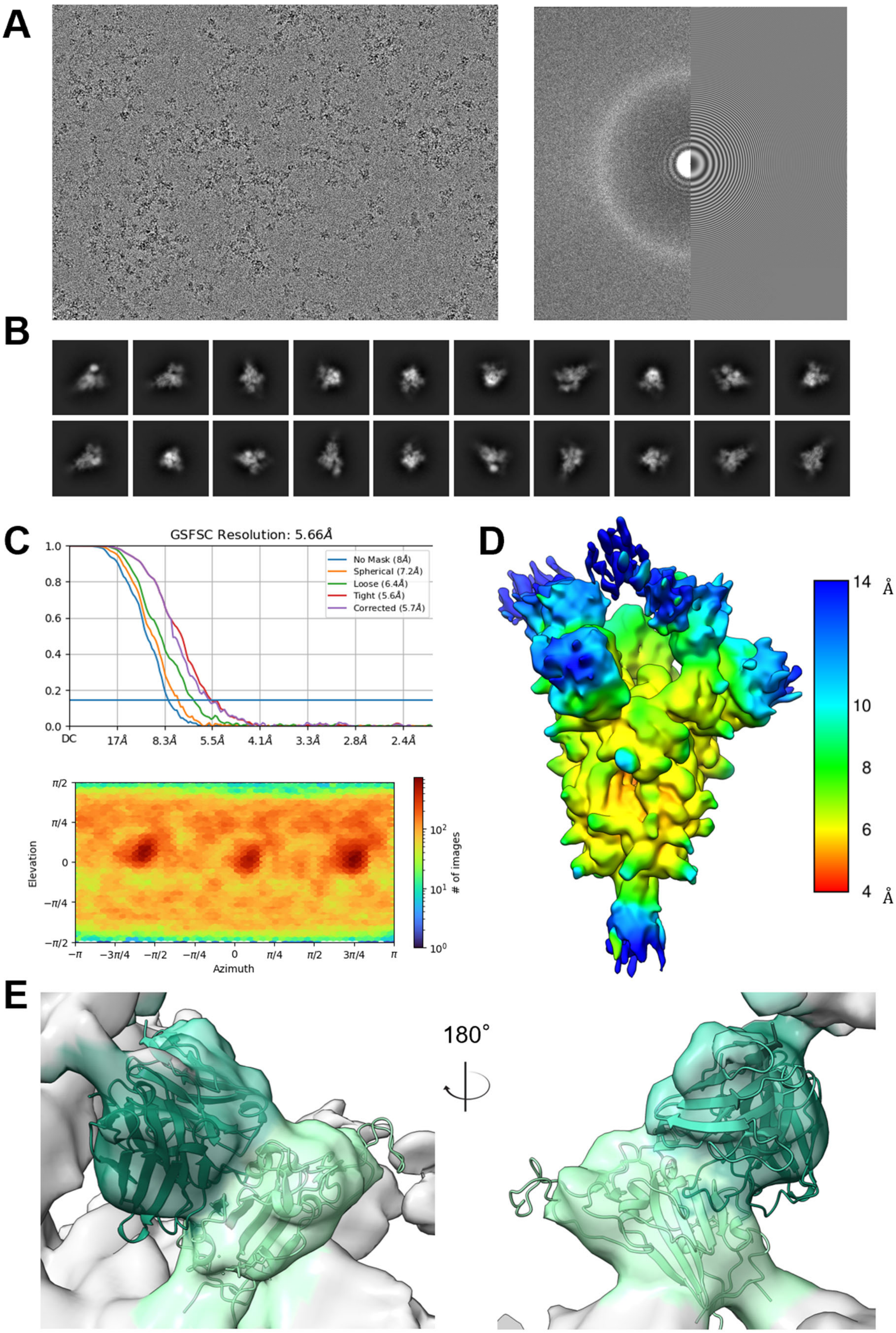
A
B
C
GSFSC Resolution: 5.66Å
No Mask (8Å)
Spherical (7.2Å)
Loose (6.4Å)
Tight (5.6Å)
Corrected (5.7Å)
1.0
0.8
0.6
0.4
0.2
0.0
DC
17Å
8.3Å
5.5Å
4.1Å
3.3Å
2.8Å
2.4Å
Elevation
Azimuth
# of images
D
14 Å
10
8
6
4 Å
E
180°

Fig. S13. Cryo-EM data processing for antibody 11-11 in complex with SARS-CoV-2 B.1.351 S trimer. (A) Representative micrograph, power spectrum, and CTF fit. **(B)** Representative 2D class averages showing S particles. **(C)** Global consensus refinement FSC curve (top panel) and particle projection viewing angle distribution (bottom panel). **(D)** Local resolution estimation mapped on surface density for global refinement. **(E)** Homology model for 11-11 Fab rigid body docked into cryo-EM map, viewed from the outside (left) and from the inside (right). The Fab is shown in teal and the RBD is shown in green.

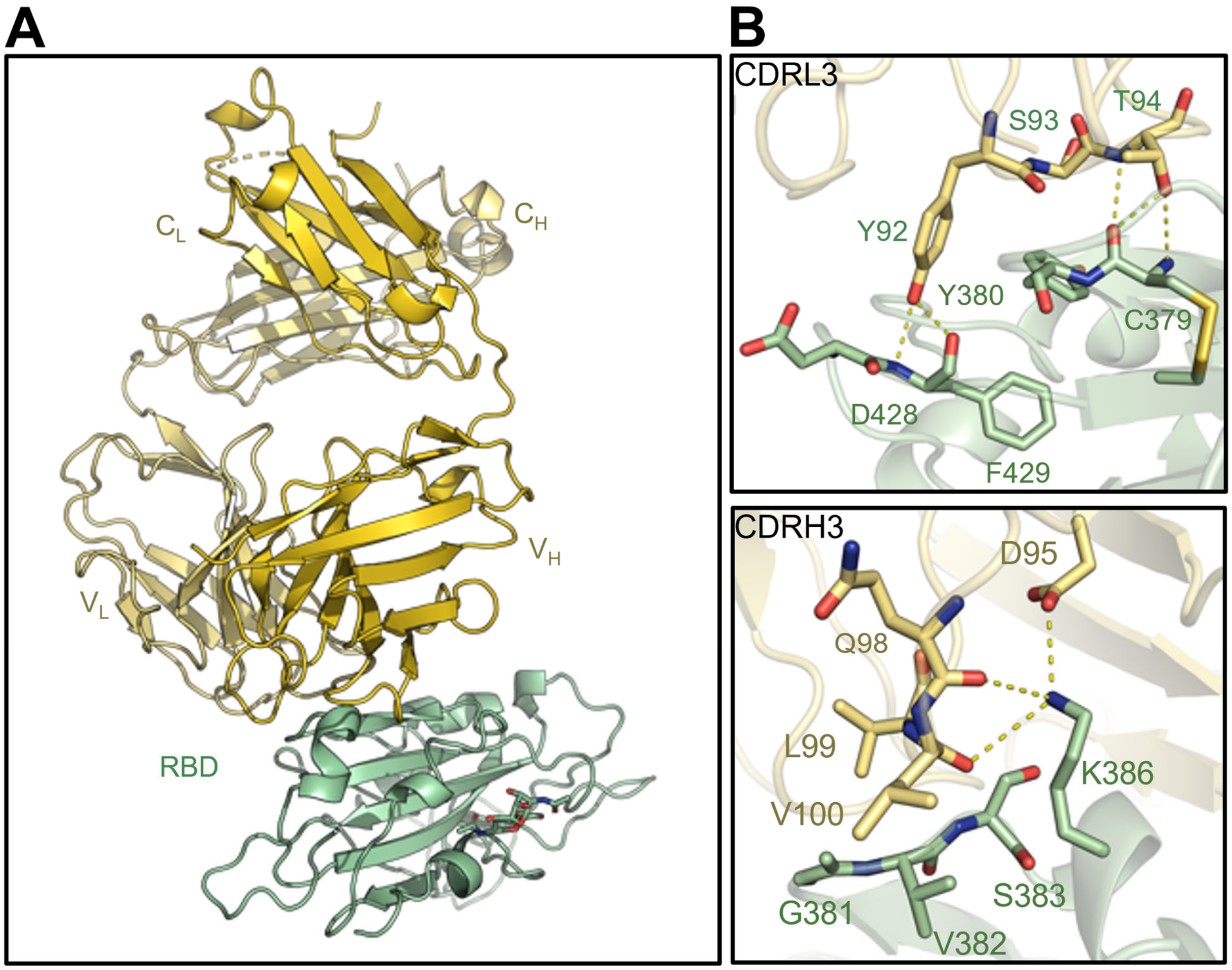

Fig. S14. Crystal structure of antibody 10-28 complexed with USA-WA1/2020 SARS-CoV-2 RBD. (A) Overview of the complex. RBD is colored in green, heavy and light chains are in dark and light yellow. **(B)** Expanded view of 10-28 interactions (dashed lines) with RBD recognition by CDRL3 (upper panel), and recognition by CDRH3 (lower panel), colored as in (A).

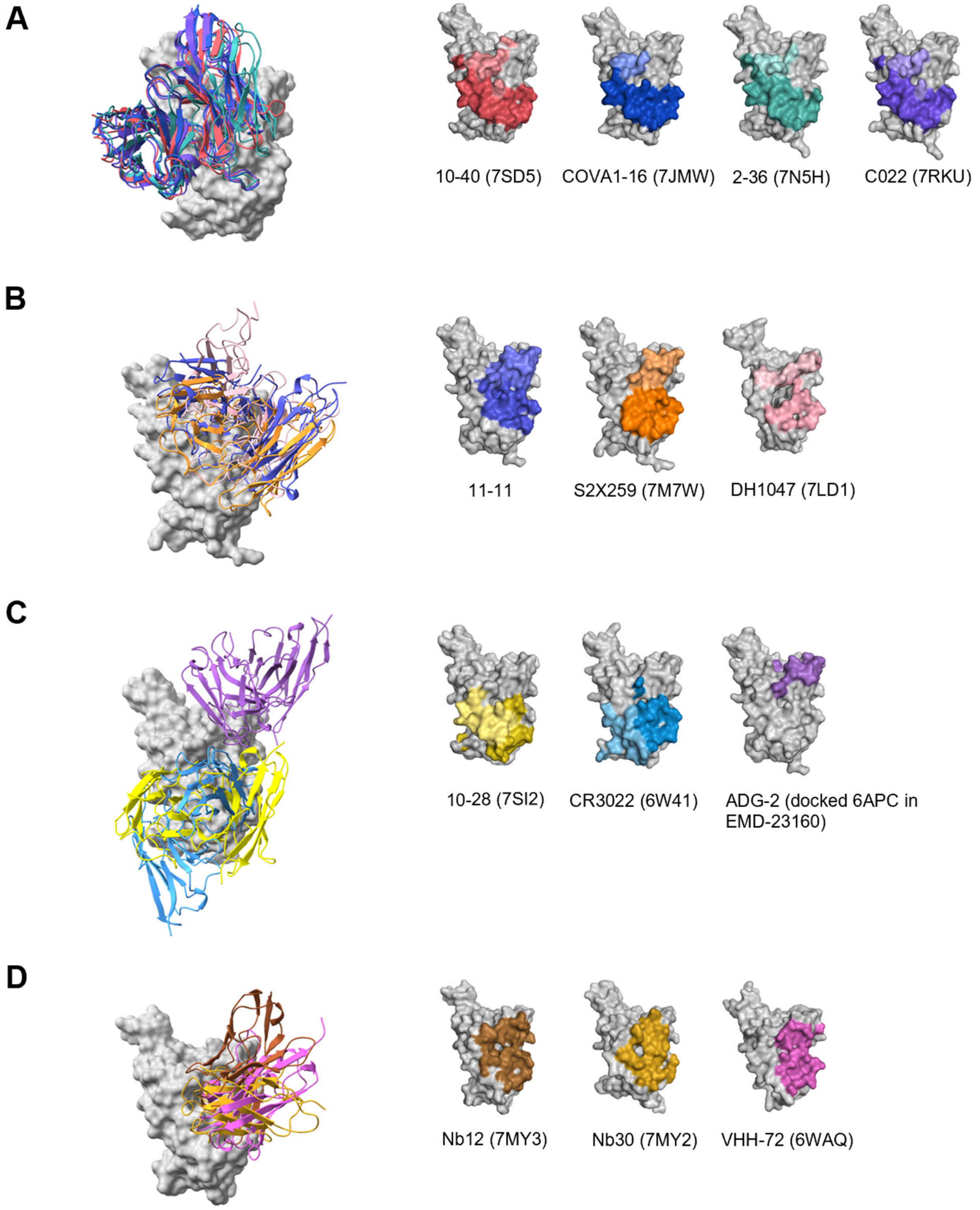

Fig. S15. Footprint comparison between 10-40, 11-11, and 10-28, and published antibodies. (A) 10-40, COVA1-16, and 2-36 recognize the inner side of the inner face of

RBD with a similar angle of approach. **(B)** 11-11, S2X259, and DH1047 recognize the outer side of the inner face of RBD with a similar angle of approach. **(C)** 10-28 and CR3022 bind to the lower middle side of the inner face of RBD. ADG-2 binds to the upper side of the inner face of RBD. Atomic coordinates were not available for ADG-2, so it was modeled by docking a homology model based on 6APC into the map EMD-23160, as described by (*11*). **(D)** Nanobodies that broadly neutralize also recognize the inner face of RBD from the outer side, similar to antibodies in (B).

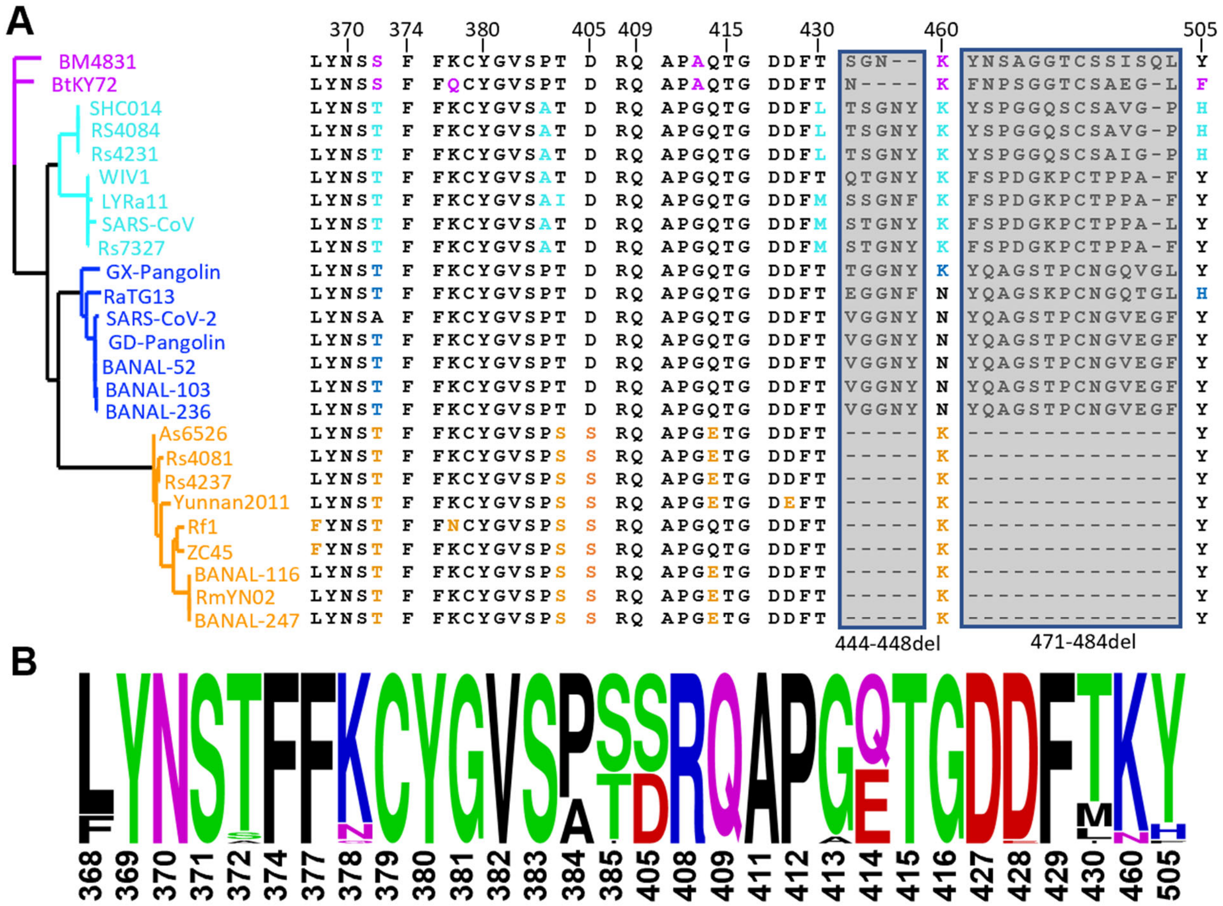

Fig. S16. 10-40 recognizes a conserved region within RBD. (A) Sequence alignment of sarbecoviruses within the epitope of 10-40. Deletions present in some sarbecoviruses are denoted in the gray boxes. **(B)** Sequence conservation of 10-40 epitope in sarbecoviruses.

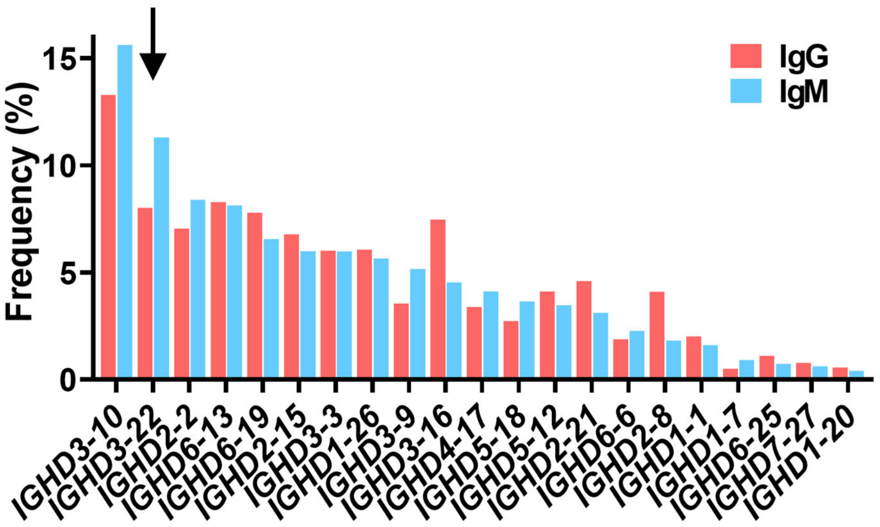

Fig. S17. D-region coding gene usage among healthy donors, ranked by frequency.

The arrow indicates *IGHD3-22*, the gene utilized by 10-40.

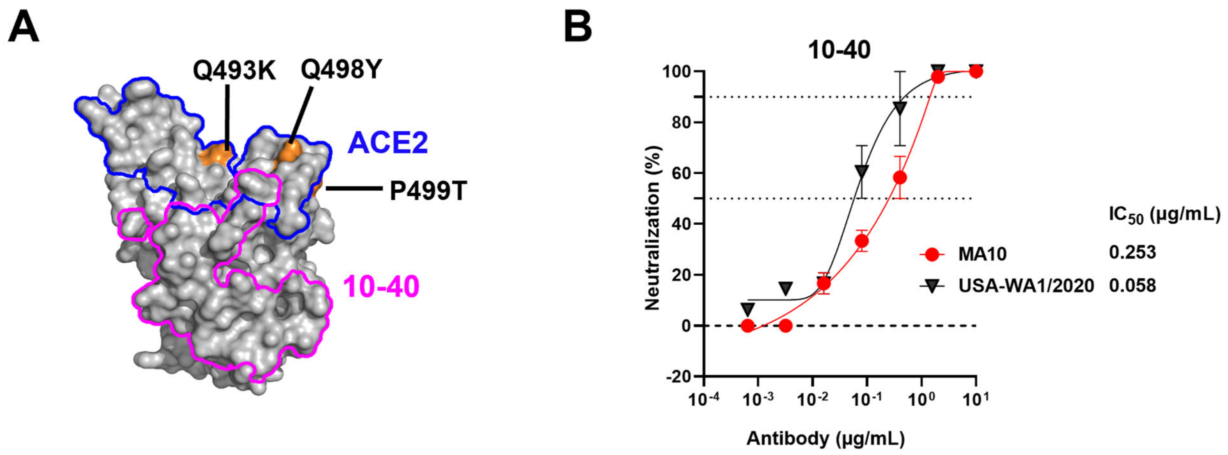

Fig. S18. Neutralization of SARS-CoV-2 MA10. (A) An overlay of the binding epitope of 10-40 (purple) and ACE2 (blue) on the RBD are shown. Mutations in the MA10 virus are denoted. **(B)** Neutralization of USA-WA1/2020 and MA10 by 10-40 in Vero-E6 cells is shown. Data are shown as mean ± SEM of threetechnical replicates. Dotted lines represent IC_{50} and IC_{90} values.

Supplementary Tables

Table S1. Cryo-EM data collection and processing.

	SARS-CoV-2 B.1.351 S2P + 10-40 Fab	SARS-CoV-2 B.1.351 S2P + 10-28 Fab	SARS-CoV-2 B.1.351 S2P + 11-11 Fab
**EMDB ID**	EMD-25146	EMD-25166	EMD-25167
**Data Collection**			
Microscope	FEI Titan Krios	FEI Titan Krios	FEI Titan Krios
Voltage (keV)	300	300	300
Magnification	81,000	81,000	81,000
Defocus Range (μm)	-0.8/-2.0	-0.8/-2.0	-0.8/-2.0
Camera	Gatan K3 BioQuantum	Gatan K3 BioQuantum	Gatan K3 BioQuantum
Pixel Size (Å/pix)	1.07	1.07	1.07
Recording Mode	counting	counting	counting
Total Dose (e-/Å^2)	42	42	42
Dose Rate (e-/pixel/s)	20	20	20
Exposure Time (s)	2.4	2.4	2.4
Frame Time (ms)	50	50	50
Software	SerialEM	SerialEM	SerialEM
**Data Processing**			
Software	cryoSPARC v3.2.0	cryoSPARC v3.2.0	cryoSPARC v3.2.0
Micrographs used	9,881	2,542	2,565
Number of Particles	799,933	251,871	281,235
Particles/Micrograph	81.0	99.1	109.6
Symmetry	C1	C1	C1
Box Size (pix)	440	384	384
Global Map FSC0.143 (Å)	3.5 / 3.6	5.2 / 6.5	5.6 / 6.4

Table S2. X-ray diffraction data collection and refinement statistics for 10-40 and 10-28 Fabs in complex with SARS-CoV-2 RBD.

	**SARS-CoV-2 RBD in complex with 10-40 Fab**	**SARS-CoV-2 RBD in complex with 10-28 Fab**
PDB ID	7SD5	7SI2
Data Collection		
Space group	$P2_12_12_1$	$C222_1$
Cell constants	75.7 79.8 118.8	123.7 226.6 186.2
Resolution range (Å)	47.67 - 1.50 (1.7-1.5)*	108.6 - 3.0 (3.1- 3.0)*
Total reflections	221728 (17750)	86848 (8514)
Unique reflections	111815 (9153)	43474 (4270)
Completeness (%)	99.28 (98.96)	97.74 (94.91)
Redundancy	2.0 (1.9)	2.0 (2.0)
I/σ	17.99 (0.89)	6.22 (1.29)
R-merge	0.016 (0.83)	0.085 (0.67)
CC1/2	1.0 (0.35)	0.69 (0.40)
Refinement		
Resolution range (Å)	47.67-1.5	88.03 - 3.2
Number of complexes per asymmetric unit	1	3
Rwork/Rfree	0.17/0.19	0.20/0.26
Number of atoms		
Protein	4905	14280
Ligands	28	84
Water	627	0
B-factors ($Å^2$)		
Protein	30.7	77.9
Ligands	74.3	77.7
Water	38.3	-
R.M.S deviations		
Bond lengths (Å)	0.013	0.012
Bond angles (°)	1.33	1.64
Ramachandran statistics		
Favored (%)	97.6	93.85
Allowed (%)	2.4	6.15
Outliers (%)	0	0

*Statistics for the highest-resolution shell are shown in parentheses.

Table S3. X-ray diffraction data collection and refinement statistics for 10-40 Fab in complex with SHC014, RaTG13, and WIV1 RBDs.

	**SHC014 RBD in complex with 10-40 Fab**	**RaGT13 RBD in complex with 10-40 Fab**	**RaGT13 RBD in complex with 10-40 Fab**
PDB ID	7TTM	7TTX	7TTY
Data Collection			
Space group	$P2_1$	$P2_12_12_1$	$P2_1$
Unit cell dimensions			
a,b,c (Å)	72.4 69.2 81.8	74.1 76.8 117.6	51.3 69.5 95.2
a,b,g (°)	90.0, 103.0, 90.0	90.0, 90.0, 90.0	90.0, 105.6, 90.0
Resolution range (Å)	79.5 – 2.2 (2.3-2.2)*	64.3-2.8 (2.9-2.8)*	55.4 - 3.1 (3.2- 3.1)*
Total reflections	260174 (23155)	224817 (22418)	79153 (7789)
Unique reflections	37573 (3698)	17042 (1656)	11715 (975)
Completeness (%)	99.2 (98.96)	99.29 (99.88)	97.6 (82.4)
Redundancy	6.9 (6.3)	13.2 (13.5)	2.0 (2.0)
I/s(I)	8.89 (0.79)	10.11 (1.43)	6.8 (6.6)
Rmerge	0.016(0.83)	0.018(1.84)	0.085 (0.67)
CC1/2	0.998 (0.598)	0.996 (0.673)	0.934 (0.40)
Refinement			
Resolution range (Å)	79.55-2.24	64.3-2.8	55.4 - 3.1
Number of complexes per asymmetric unit	1	1	1
Rwork/Rfree	0.20/0.25	0.22/0.29	0.22/0.28
Number of atoms			
Protein	638	635	633
Ligands	42	15	28
Water	109	-	0
B-factors			
Protein	56.79	81.05	81.4
Ligands	74.3	80.91	81.1
Water	52.01	-	
R.m.s. deviations			
Bond lengths (Å)	0.010	0.014	0.014
Bond angles (°)	1.32	1.73	1.97
Ramachandran statistics			
Favored (%)	96.34	92.03	96.24
Allowed (%)	3.66	7.77	3.76
Outliers (%)	0	0	0

*Statistics for the highest-resolution shell are shown in parentheses.

Table S4. Signature of 10-40-like reproducible antibody class.

Region	Signature motifs	Compatible genes	Critical
**CDRH1**	GGS[IV]SSS[NR][FY]Y	*IGHV4-39, IGHV4-61, IGHV4-31, IGHV4-30-2/4*	No
	GYTFTSYY	*IGHV1-46, IGHV1-2*	No
**CDRH3**	ARx{4-5}YYDRSGYx{8-9}	*IGHD3-22*01*	Yes
**Light Chain**	NA	*IGLV6-57, IGKV3-20, IGKV1-33, IGKV1-9*	No

Table S5. Accession numbers for sarbecovirus sequences used in this study.

ID	Accession	Source
SARS-CoV-2	MN908947	NCBI
Pangolin_GD	MT799524	NCBI
RaTG13	MN996532	NCBI
Pangolin_GX	MT040333	NCBI
SARS-CoV-1_Urbani_HP03	AY278741	NCBI
SARS-CoV-1_BJ02_HP03M	AY278487	NCBI
SARS-CoV-1_HGZ8L1-A_HP03E	AY394981	NCBI
SARS-CoV-1_GD01_HP03L	AY278489	NCBI
SARS-CoV-1_GZ-C_HP03L	AY394979	NCBI
SARS-CoV-1_Sino1-11_HP03L	AY485277	NCBI
SARS-CoV-1_Sin852_HP03L	AY559082	NCBI
SARS-CoV-1_SZ3_PC03	AY304486	NCBI
SARS-CoV-1_SZ13_PC03	AY304487	NCBI
SARS-CoV-1_SZ1_PC03	AY304489	NCBI
SARS-CoV-1_GD03T0013_HP04	AY525636	NCBI
SARS-CoV-1_GZ0402_HP04	AY613947	NCBI
SARS-CoV-1_PC4-127_PC04	AY613951	NCBI
SARS-CoV-1_PC4-137_PC04	AY627045	NCBI
SARS-CoV-1_PC4-13_PC04	AY613948	NCBI
WIV16	KT444582	NCBI
WIV1	KF367457	NCBI
Rs7327	KY417151	NCBI
LYRa11	KF569996	NCBI
Rs4231	KY417146	NCBI
RsSHC014	KC881005	NCBI
Rs4084	KY417144	NCBI
BM48-31	NC014470	NCBI
BtKY72	KY352407	NCBI
ZXC21	MG772934	NCBI
ZC45	MG772933	NCBI
JL2012	KJ473811	NCBI
Rf1	DQ412042	NCBI
HeB2013	KJ473812	NCBI
273-2005	DQ648856	NCBI
Rf4092	KY417145	NCBI
YN2013	KJ473816	NCBI
RmYN02	EPI_ISL_412977	GISAID
As6526	KY417142	NCBI
Rs4237	KY417147	NCBI
Rs4081	KY417143	NCBI
Rp3	DQ071615	NCBI
279-2005	DQ648857	NCBI
Shaanxi2011	JX993987	NCBI
Yunnan2011	JX993988	NCBI
Rs4247	KY417148	NCBI
HKU3-13	GQ153548	NCBI
HKU3-1	DQ022305	NCBI
GX2013	KJ473815	NCBI
Longquan-140	KF294457	NCBI
HKU3-8	GQ153543	NCBI
HuB2013	KJ473814	NCBI
Hp-BCoV_Zhejiang_2013	KF636752	NCBI
